# Perturbed pediatric circulating metabolome in mild and severe dengue disease

**DOI:** 10.1128/jvi.01572-25

**Published:** 2025-10-29

**Authors:** Paul S. Soma, Rebekah C. Gullberg, Barbara Graham, M. Nurul Islam, Guillermina Kuan, Angel Balmaseda, Carol D. Blair, Barry J. Beaty, John T. Belisle, Eva Harris, Rushika Perera

**Affiliations:** 1 Department of Microbiology, Immunology, and Pathology, Colorado State University 3447, Fort Collins, Colorado, USA; 2 Department of Chemistry, Biochemistry and Physics, South Dakota State University 2019, Brookings, South Dakota, USA; 3 Sustainable Sciences Institute, Managua, Nicaragua; 4 Centro de Salud Sócrates Flores Vivas, Managua, Nicaragua; 5 Laboratorio Nacional de Virología, Centro Nacional de Diagnóstico y Referencia, Ministry of Health 217874 , Managua, Nicaragua; 6 Division of Infectious Diseases and Vaccinology, School of Public Health, University of California Berkeley 1438, Berkeley, California, USA; Wake Forest University School of Medicine, Winston-Salem, North Carolina, USA

**Keywords:** hemorrhagic fever, fever, dengue, lipidomics, metabolomics, serum, pediatric, severe disease, infectious disease, biomarker

## Abstract

Four billion people are at risk of infection with dengue viruses (DENV), and this burden is rapidly increasing due to geographic expansion of the mosquito vector. Infection with any of the four serotypes of DENV can result in a self-limiting but debilitating febrile illness (DF), and some infections progress to severe disease with hemorrhagic manifestations and shock (dengue hemorrhagic fever/dengue shock syndrome [DHF/DSS]). DENV infection drives the metabolic state of host cells for viral benefit and induces a host-immune response with metabolic implications that link to disease. Here, a dynamic metabolic response to DENV infection and disease was measured in 535 pediatric patients from Nicaragua using liquid chromatography-tandem mass spectrometry. Metabolomic analyses revealed profound disruptions of critical biochemical pathways and metabolites within the circulating metabolome, especially in those with more severe manifestations of dengue disease. A biomarker panel of 28 metabolites was utilized to classify DF versus DHF/DSS with high sensitivity and specificity, equating to a balanced accuracy of 96.88%. Identified metabolites belonged to biochemical pathways of omega-3 and omega-6 fatty acids, sphingolipids, dipeptides, purines, and tryptophan metabolism. Dipeptides emerged as the most critical molecules for severe disease classification. Additionally, a previously reported trend between serotonin and platelets in DHF patients was expanded upon here, revealing a major depletion of serotonin, but not platelets, in DSS patients. In this study, the perturbed metabolome was used for disease state classification and exploration of the biochemistry of severe dengue disease pathology.

**IMPORTANCE:**

The international burden of dengue is intensifying, as the number of reported cases in only the first 5 months of 2025 exceeded that of the previous annual high in 2023. The occurrence of deadly severe manifestations of dengue disease will escalate as the total cases rise, and pediatric patients are at greater risk of developing the rapidly progressing severe dengue diseases than adults. Suboptimal vaccines, lack of clinically approved therapeutics, and no methodologies for prognosis of severe disease exacerbate the difficulty of preventative and supportive care. Because human metabolism is rapidly altered due to infection, perturbations in patients’ circulating metabolome can be attributed to dengue disease and correlated to severity. This study contributes metabolic biomarkers of dengue disease in pediatric patients from Nicaragua, indicating that metabolic biomarkers are conserved across patients of different ages and geographic and genetic backgrounds. With validation across many cohorts, there is potential to improve diagnostics.

## INTRODUCTION

Dengue viruses (DENV) are mosquito-borne flaviviruses that place 3.97 billion people at risk of infection each year, with up to 390 million infections estimated annually, rendering them the most prevalent arboviruses worldwide (1–3). In the first 5 months of 2024, there were 7.6 million reported dengue cases, including 16,000 cases of severe disease and 3,000 deaths—quickly surpassing the previous annual high of 4.6 million dengue cases in 2023 (4). There are four DENV serotypes (DENV1–4). While infection with one serotype can cross-protect from infection with a heterologous serotype in the short term, it can lead to antibody-dependent enhancement of disease in the longer term (5). DENV is the etiologic agent of dengue fever (DF), which is an incapacitating but self-limited disease; however, some cases progress to dengue hemorrhagic fever (DHF) or the potentially fatal dengue shock syndrome (DSS). Peak viremia and fever/symptom onset occur several days after the human host is bitten by an infected mosquito. The acute phase lasts 7 days and includes the “critical phase,” which occurs 4 to 7 days after fever onset (6). Most patients present with DF and defervesce during the critical phase. In a minority of cases, severe manifestations present during the critical phase, with the hallmark vascular leakage that can lead to shock and lethal outcomes. Various proteins, peptides, and metabolites in adult patients have been previously proposed as biomarkers for severe dengue disease (7–14). Metabolite biomarkers hold notable advantages, and the study of metabolism presents an opportunity to help address challenges in biomarker discovery and in triaging rapidly progressing diseases such as dengue.

Metabolites are the small molecule intermediates and products of biochemical reactions in host cells. Metabolic reactions are driven by upstream gene expression (transcription and translation) and enzyme activity, as well as by the current cellular environment, including active metabolic signaling and bystander effects (15–17). The metabolome is known as an effector of phenotype because it is dynamic and responds quickly to external stimuli (18). Therefore, the metabolome may describe the current physiological state of the system under study with greater temporal resolution than upstream biomolecules. Perturbations in cellular metabolism of host tissues due to viral infection and the host immune response are reflected in the host’s circulating metabolite profile. Accordingly, measuring the circulating metabolome of DENV-infected patients can provide information about system-wide metabolic shifts upon DENV infection and onset of disease.

Infection with DENV prompts perturbations in host cellular metabolism to facilitate various stages of the viral lifecycle and induces a host immune response that is also associated with metabolic alterations (11–13, 19, 20). Metabolomic measurements that lead to mapping the dysregulated pathways in DENV infection and disease can improve understanding of pathogenic processes; define enzyme drug targets that, when inhibited, interfere with the viral lifecycle; or identify biomarkers of severe dengue disease that aid in triage. In this study, the DENV infection-induced metabolic perturbations in pediatric patients were measured using liquid chromatography-tandem mass spectrometry (LC-MS/MS). The measured perturbed metabolome was used for disease state classification, metabolic pathway analysis, and the exploration of the biochemistry of severe dengue disease pathology.

## RESULTS

### Clinical samples

**Fig 1:**
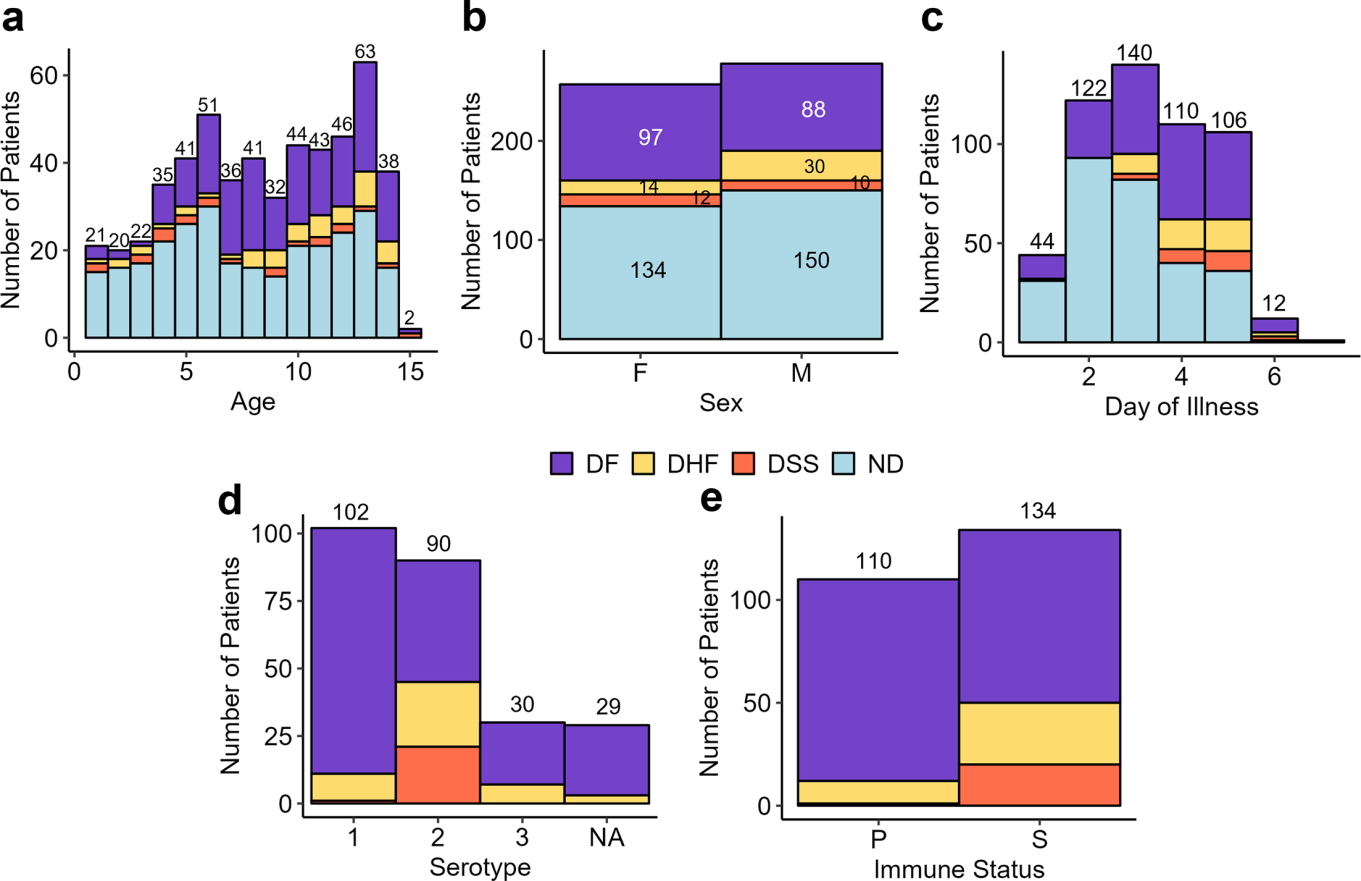
Summary of selected pediatric patient metadata. (**a**) Patient age, (**b**) sex, (**c**) day of illness at enrollment, (**d**) DENV serotype, and (**e**) immune status; primary (P), secondary (S), non-applicable (NA). Numbers above the bars represent the total number of patients in each group. Numbers within each color bar for sex represent the number of patients with the respective disease outcome.

This study included 535 retrospective serum and plasma samples from individual pediatric patients enrolled in two well-established studies in Managua, Nicaragua. Selected patient demographics are summarized in Fig. 1a through e. Statistical assessment of patient demographics and distribution of sample types between disease states (clinical diagnosis) is included in Table S1. Of the 535 suspected dengue cases, 251 were laboratory-confirmed as dengue-positive, and 284 were non-dengue (ND) febrile illnesses. Based on the 1997 World Health Organization disease severity criteria (21), the dengue cases were classified as DF (185), DHF (44), and DSS (22). The study included pediatric patients of ages 1 to 15 years (Fig. 1a) and similar numbers of females (*n* = 257, 48%) and males (*n* = 278, 52%) (Fig. 1b). All samples used for this study were collected on days 1 to 7 of illness (Fig. 1c). Dengue patients were positive for DENV serotypes 1, 2, or 3, or had an undetermined serotype (Fig. 1d). Immune status (primary or secondary infection) was reported (Fig. 1e).

### Metabolic pathway analysis enables insight into dengue disease pathology

**Fig 2:**
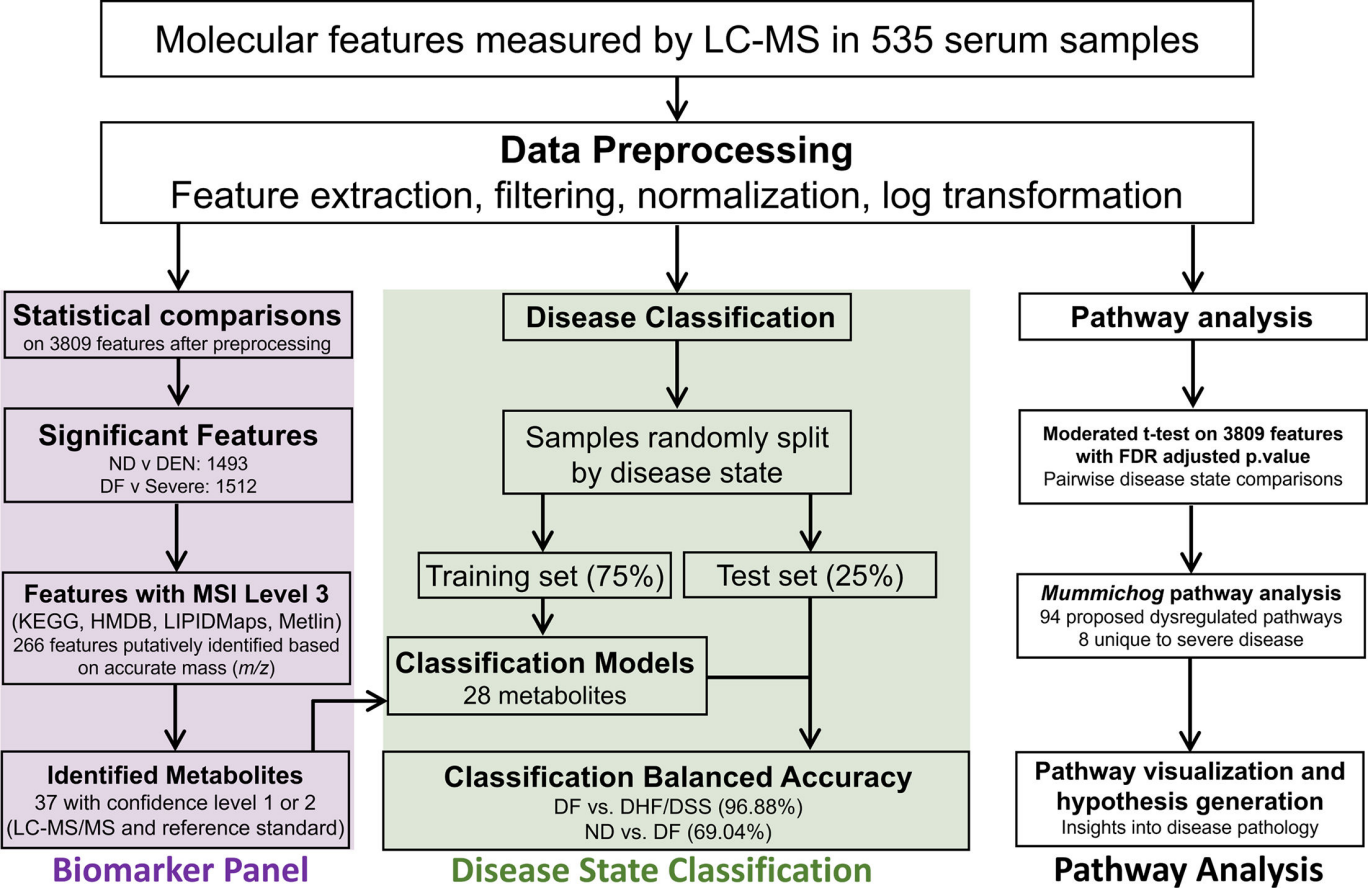
Data processing and analysis workflow and summarized results for the biomarker panel, disease state classification, and pathway analysis.

The circulating metabolome of each patient was measured via untargeted LC-MS/MS, and the final feature list after data preprocessing contained 3,809 features (Fig. 2). For hypothesis generation and to glean biological information from this metabolomics data set, all 3,809 molecular features (RT, *m/z*, p-value, and t-score) were fed into the *mummichog* algorithm to be tentatively identified and mapped to known metabolic pathways. Pathway analysis using all molecular features proposed 94 tentatively enriched metabolic pathways, each pathway having its own magnitude and direction of dysregulation and statistical significance between three pairwise comparisons: DF vs DHF, DF vs DSS, and DHF vs DSS. Of the 94 pathways, 86 were common among the three comparisons, 3 were unique to DF vs DHF, and 5 were unique to DHF and DSS. Though 86 pathways were identified to be altered in all disease states, the extent of perturbation between comparisons was different. Median t-scores and Fisher’s exact p-values for all pathways and comparisons are shown in Table S2.

**Fig 3:**
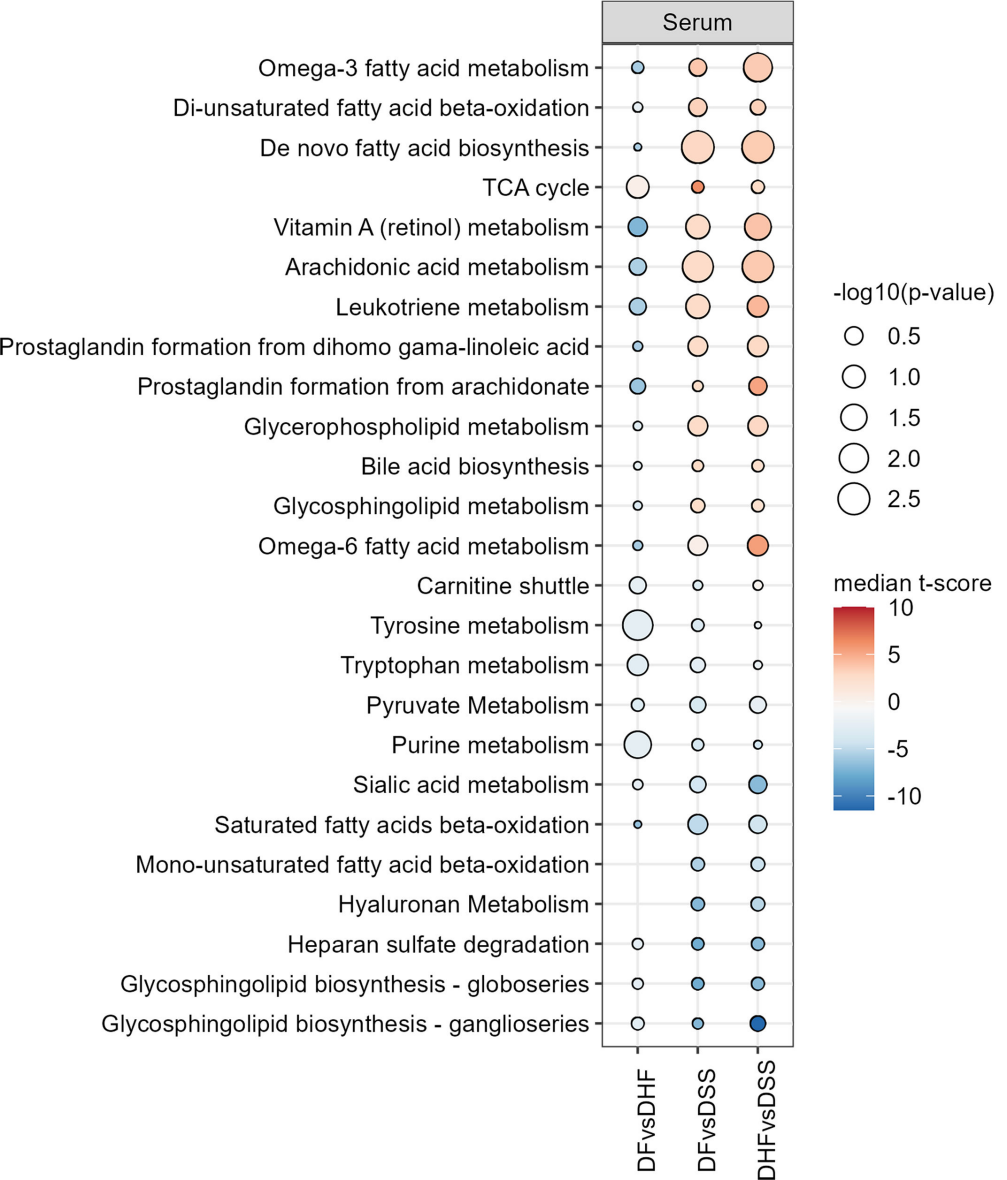
Dysregulation of selected pathways in dengue disease. Red indicates the pathway is upregulated, and blue indicates the pathway is downregulated, in the more severe form of the disease. Dysregulation direction and magnitude are based on the median t-score of all dysregulated metabolites in the indicated pathway. Statistical significance is represented by the log_10_(p-value) from Fisher’s exact test.

Twenty-five perturbed pathways with known biological relevance to dengue and other pathological phenotypes are shown in Fig. 3. The 25 pathways are related to the metabolism of tryptophan, lipids (e.g., fatty acids, phospholipids, eicosanoids, and sphingolipids), bile acids, amino acids, purines, sugars, and other cellular energy-related small molecules. The TCA cycle, which drives cellular energy generation, was upregulated in DSS, and pathways related to amino acids and purines were downregulated. Dysregulation of lipids, notably upregulation of fatty acid (FA) synthesis during DENV infection, aligns with our previous *in vitro* and *in vivo* studies (10, 20). Metabolism of bile acids and retinoic acid was upregulated in DSS, which may relate to liver damage observed in DHF/DSS (22, 23). Regarding sugars, hyaluronan metabolism, sialic acid metabolism, and heparan sulfate degradation were all downregulated in DSS, which may relate to the endothelial glycocalyx and its role in viral entry (24, 25) or its breakdown in the loss of vascular integrity in severe dengue disease (26–29). The results of pathway analysis were used to inform metabolic pathway and metabolite targets for molecular structure identification and biomarker panel development.

### Circulating metabolites are perturbed in dengue disease pathology

The workflow to identify molecular features of significant differential abundance across disease states is shown in Fig. 2. A total of 1,512 features revealed significant (adjusted *P*-value < 0.05) differential abundance for at least one of three pairwise disease state comparisons: DF vs DSS, DHF vs DSS, or DF vs DHF. Volcano plots were used to visualize the differential abundance of features between disease states (Fig. S1), and histograms were used to visualize the frequency of feature differential abundance (Fig. S2) (Figure S2). In the pairwise comparison of DF vs DSS, 1,117 and 160 features were identified as less abundant (log_2_FC < −1) and more abundant (log_2_FC > 1), respectively, in DSS, and 235 features with significant p-values presented with −1 < log2 FC <1 and were considered unperturbed. Comparing DHF vs DSS resulted in 961 and 320 features that were less or more abundant, respectively, in DSS, with 231 features considered unperturbed. Comparing DF vs DHF resulted in 364 features that were less abundant in DHF, 31 features that were more abundant in DHF, and 1,117 that met the defined criteria for “unperturbed.”

The 1,512 significant features possess information that can differentiate disease state but are not clinically useful without being identified. Therefore, to develop a tool with potential clinical utility, a subset of molecules was identified. These molecules were chosen based on pathway analysis, stark differential abundance between disease states, known relevance to DENV infection and disease, and previously published literature. Twenty-eight metabolites were included in the biomarker panel, all of which were identified using analytical standards and/or LCMS/MS and had an adjusted *P*-value < 0.05 for at least one pairwise dengue disease state comparison. Twelve and 16 metabolites were identified at confidence levels 1 and 2, respectively (30). A total of 48 relevant metabolites were identified at confidence levels 1 through 3, with level 1 representing the highest confidence and level 3 indicating tentative molecular formula prediction based on accurate mass (*m/z*). Serum metabolites were identified at confidence level 1 by comparing LC-MS/MS metrics to pure, synthetic reference standards, and at confidence level 2 by comparing experimental collision-induced dissociation data to known dissociation pathways and MS/MS spectral libraries. Identification of metabolites at each confidence level is further described in the methods and previous literature (30). The LC-MS/MS data used to validate metabolite identities at confidence level 1 are provided in Fig. S3a through n. Further details and relevant pathways for all metabolites identified in this study are summarized in Table S3 and S4 summarizes the log2FC and adjusted p-values from a moderated *t*-test for the pairwise disease state comparisons of these metabolites.

Circulating creatinine level was used as a quality control in this study. Creatinine is well known to positively correlate with increasing age (31), and this trend was recapitulated in the current data set. Creatinine was identified in the current data set, and a linear regression on creatinine abundance as a function of patient age produced a positive slope, independent of disease state and sex (Fig. S4). Other metabolites identified did not strongly correlate with age.

### Identified metabolites accurately classify severe dengue disease

**Fig 4:**
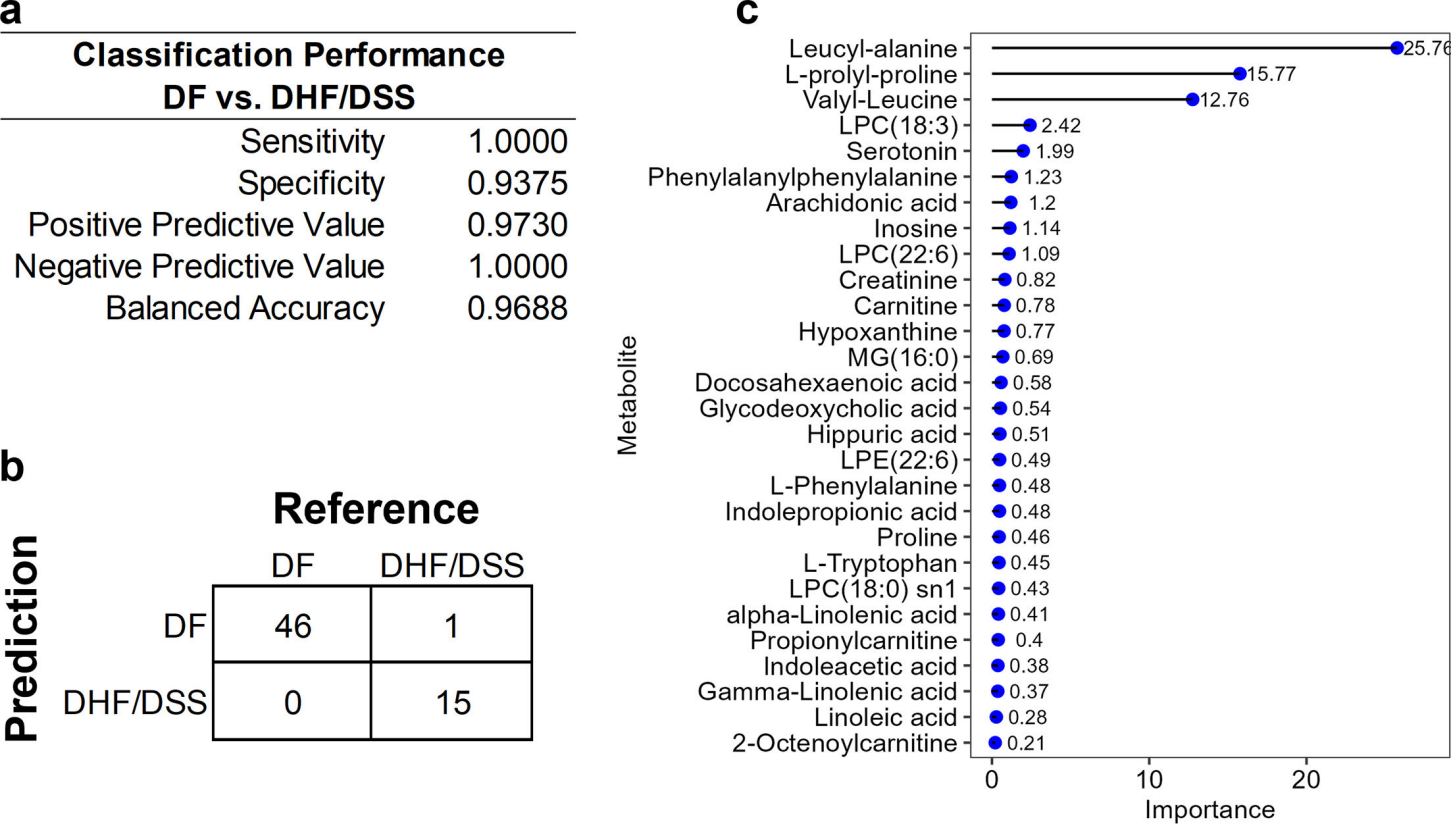
Metabolite biomarker panel accurately classifies dengue disease state. (**a**) Random forest classification performance metrics for DF vs DHF/DSS. Sensitivity is the proportion of correctly classified DF samples. Specificity is the proportion of correctly classified DHF/DSS samples. Balanced accuracy is the average of sensitivity and specificity. Positive predictive value is the proportion of samples classified as DF that are truly DF. Negative predictive value is the proportion of samples classified as DHF/DSS that are truly DHF/DSS. (**b**) Confusion matrix for the classification of DF from DHF/DSS. (**c**) Random forest variable importance plot.

We evaluated the capacity of the biomarker panel for disease state classification. Using 28 identified metabolites, an ensemble of classification models was trained and tested on their ability to differentiate DF from DHF/DSS or ND from DF. A random forest (rf) model performed exceedingly well in differentiating DF from DHF/DSS, resulting in a balanced accuracy of 96.88% (Fig. 4a). When challenged with the classification of the test set, rf misclassified only one DHF/DSS sample as DF (Fig. 4b). The variable importance plot for DF vs DHF/DSS classification is displayed in Fig. 4c, indicating the impact of each metabolite on the quality of disease state classification. The high classification accuracy of the rf model was an important outcome, but underprediction of severe disease was not satisfactory. To investigate if the day of illness was a confounding variable for classification of DF vs DHF/DSS, we performed a subset analysis for samples collected on days 3 to 6 of illness. This subset classification analysis correctly classified all samples (100% balanced accuracy), and the single DHF/DSS sample was no longer underpredicted. Further, when plasma samples were excluded from classification, all samples were correctly classified besides one DHF/DSS, and even the days 3 to 6 subset analysis did not properly classify that single sample. For the classification of ND vs DF, performance was poorer, resulting in a balanced accuracy of 69.04% (Fig. 2).

### Dipeptides contribute greatly to classification success

Various dipeptides and amino acids were detected in patient samples (Fig. S7). The dipeptides L-prolyl-proline, valyl-leucine, and leucyl-alanine were major drivers of disease state classification and were depleted in all DSS patients and in some DHF patients, in whom a bimodal distribution of abundance was observed. The amino acid proline was significantly decreased in DHF and DSS patients compared to DF. Phenylalanyl-phenylalanine circulating levels also significantly decreased with dengue disease severity, but the amino acid phenylalanine was unperturbed across disease states.

### Tryptophan metabolism is reduced in dengue disease

**Fig 5:**
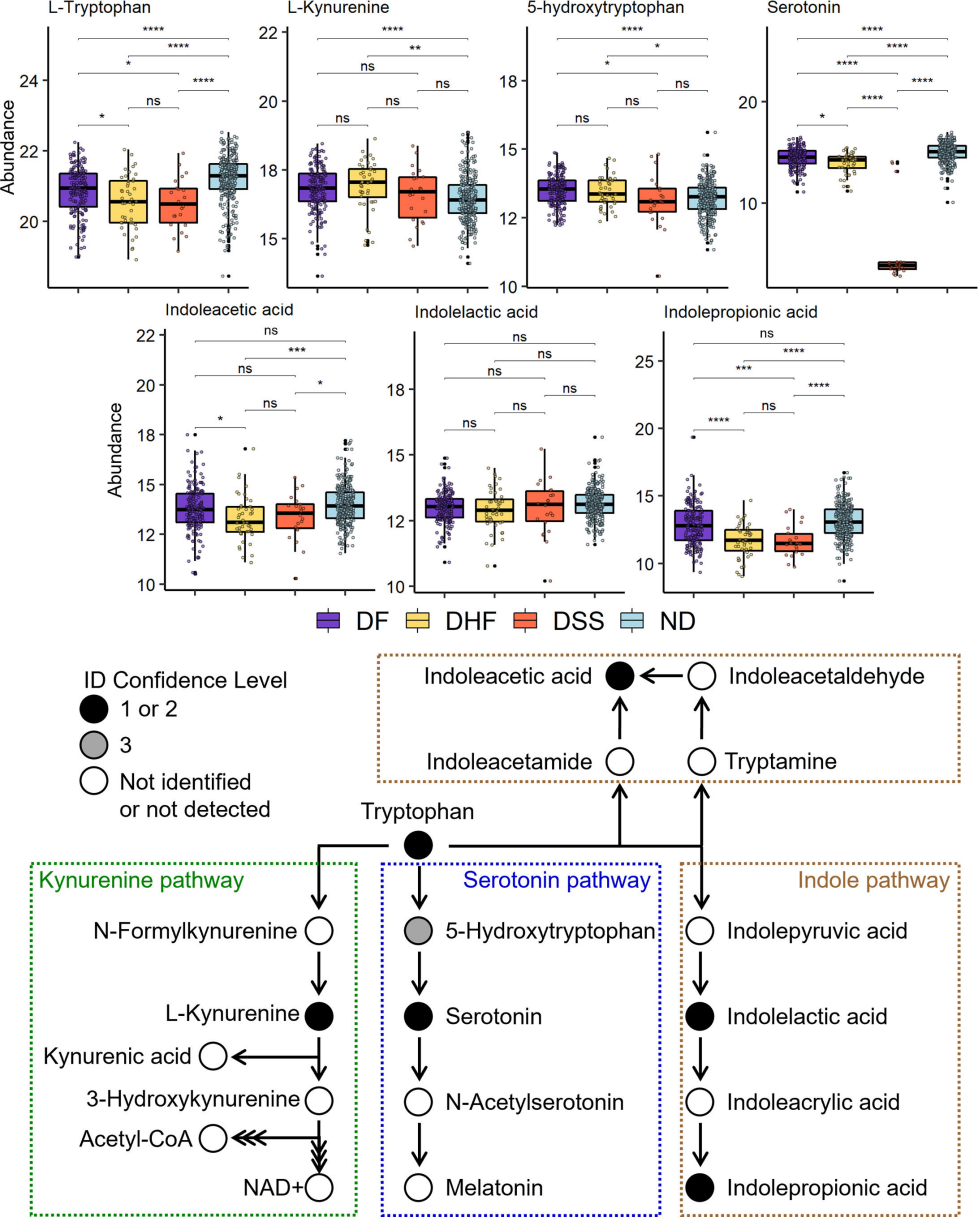
Tryptophan metabolism is perturbed in dengue disease. (Top) Abundance of tryptophan metabolites in ND and dengue patients. Abundance is represented as the normalized and log2-transformed LC-MS peak area. Benjamini-Hochberg adjusted p-values generated using a moderated *t*-test were used to define statistical significance level; where ns is not significant and *P* < 0.0001, 0.001, 0.01, and 0.05 are each represented by ****, ***, **, and *, respectively. (Bottom) Eight metabolites from three tryptophan metabolic pathways were measured in this data set. The color of each dot in the pathway corresponds to the identification confidence level, where 1 is the highest and 3 is tentative identification. Triple arrows between metabolites indicate that multiple metabolic steps are required.

Tryptophan metabolism has been implicated in many disease conditions, including viral infection. In this study, tryptophan and seven of its metabolites were identified in patient samples. Metabolite abundance in each patient sample was stratified by disease state and visualized using boxplots, and tryptophan metabolic pathways were illustrated (Fig. 5). Additionally, the differential abundance of each metabolite is described by log_2_FC and adjusted p-value (Table S4).

**Fig 6:**
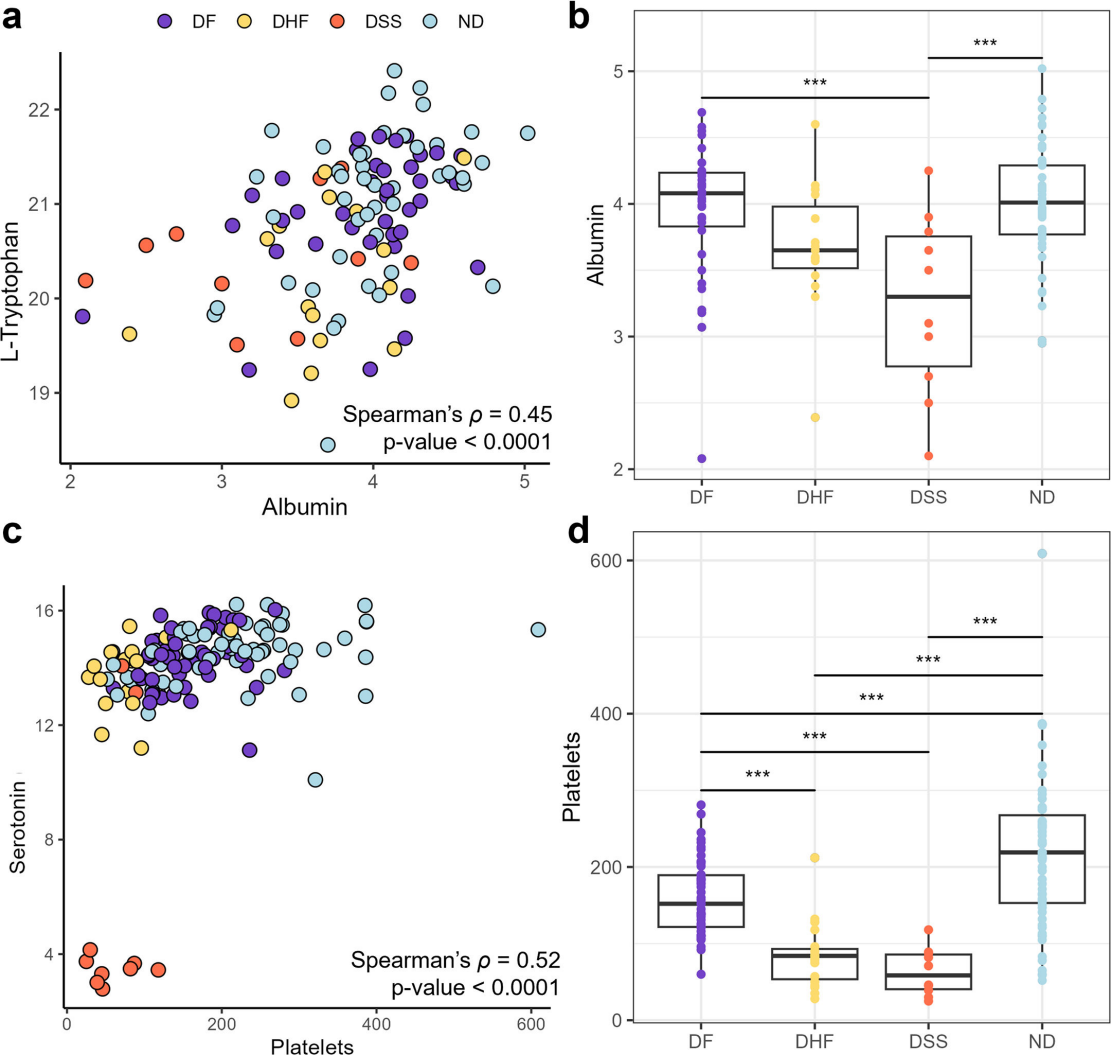
Clinical measures of albumin and platelets correlate with tryptophan metabolism and dengue disease states. Correlation of albumin levels (g/dL) with (**a**) circulating tryptophan levels (all disease states: ρ = 0.45; *P*-value < 0.0001, DF only: ρ = 0.30; *P*-value = 0.06, DHF only: ρ = 0.32; *P*-value = 0.24, DSS only: ρ = 0.60; *P*-value = 0.60, ND only: ρ = 0.45; *P*-value = 0.002) and (**b**) disease state. Correlation of platelet levels (platelets/µL) with (**c**) serotonin levels (all disease states: ρ = 0.52; *P*-value < 0.0001, DF only: ρ = 0.31; *P*-value = 0.02, DHF only: ρ = 0.30; *P*-value = 0.21, DSS only: ρ = 0.09; *P*-value = 0.80, ND only: ρ = 0.35; *P*-value = 0.005) and (**d**) disease state. *P*-values for Spearman correlations were generated via the Kruskal-Wallis test. Tukey adjusted linear models were used to define statistical significance level for boxplots; where ns is not significant and *P* < 0.0001, 0.001, 0.01, and 0.05 are each represented by ****, ***, **, and *, respectively.

Tryptophan abundance was significantly lower in DF patients compared to ND. Regarding dengue disease severity, tryptophan abundance progressively decreased in DHF and DSS patients. Because tryptophan binds to the protein albumin in circulation, sample-matched clinical measures of albumin were correlated with tryptophan levels in a subset of 144 patients. Tryptophan and albumin levels had a positive Spearman’s ρ correlation coefficient of 0.45 (*P*-value < 0.0001) (Fig. 6a). Within each individual disease state, positive correlations were observed but with variable p-values, likely due to differences in sample size. Further, albumin levels in DSS (*P*-value < 0.0003) and DHF (not significant) were lower on average than in ND or DF patients (Fig. 6b), suggesting that disease state may be the main driver of correlation.

Kynurenine, a metabolite from one of the two host-driven tryptophan metabolic pathways, was significantly elevated in DF and DHF compared to ND. Kynurenine was not significantly elevated in DSS compared to ND, and there were no observed significant differences in kynurenine between dengue disease states. The first metabolic product in the serotonin pathway, 5-hydroxytryptophan, was significantly elevated in DF and DHF, but not significantly different in DSS, compared to ND. Accordingly, 5-hydroxytryptophan was significantly lower in DSS compared to DF.

Serotonin, the downstream metabolite of 5-hydroxytryptophan, was significantly decreased in all dengue disease states. In DF and DHF patients, serotonin abundance was moderately decreased relative to ND. Strikingly, serotonin abundance was massively decreased in DSS patients. Because serotonin is known to affect platelet function and activity, for a subset of 144 patients, sample matched clinical measures of platelets were correlated to serotonin levels. Serotonin and platelet levels had a positive Spearman correlation coefficient of 0.52 (*P*-value < 0.0001) (Fig. 6c). For DSS patients only, a low correlation coefficient of 0.09 was observed, suggesting differences between disease states may be the main driver of correlation. Further, platelet levels were significantly decreased in all dengue disease states compared to ND, but platelet levels were not significantly different between DHF and DSS patients (Fig. 6d).

A trend in serotonin abundance was observed when stratified by day of illness, and a similar trend was observed for platelets for a subset of patients (Fig. S5). In a cross-sectional analysis of DF patients, serotonin abundance began “normal” or relatively high on day 1 of illness, then decreased until days 5 or 6, where the rate of decrease (slope) levels off. The same decreasing trend was observed in DHF. In DSS patients, serotonin levels were massively decreased on all days (days 3 to 6).

Three metabolic products from the indole metabolic pathway of tryptophan via the gut microbiota were measured: indoleacetic acid (IAA), indolelactic acid (ILA), and indolepropionic acid (IPA) (Fig. 5). The abundances of IAA and IPA were significantly decreased in DHF/DSS, and the abundance of IAcrA was significantly decreased in all dengue disease states (Table S4). ILA abundance was not observed to differ significantly among disease states.

### Fatty acid metabolism is elevated in severe dengue disease

**Fig 7:**
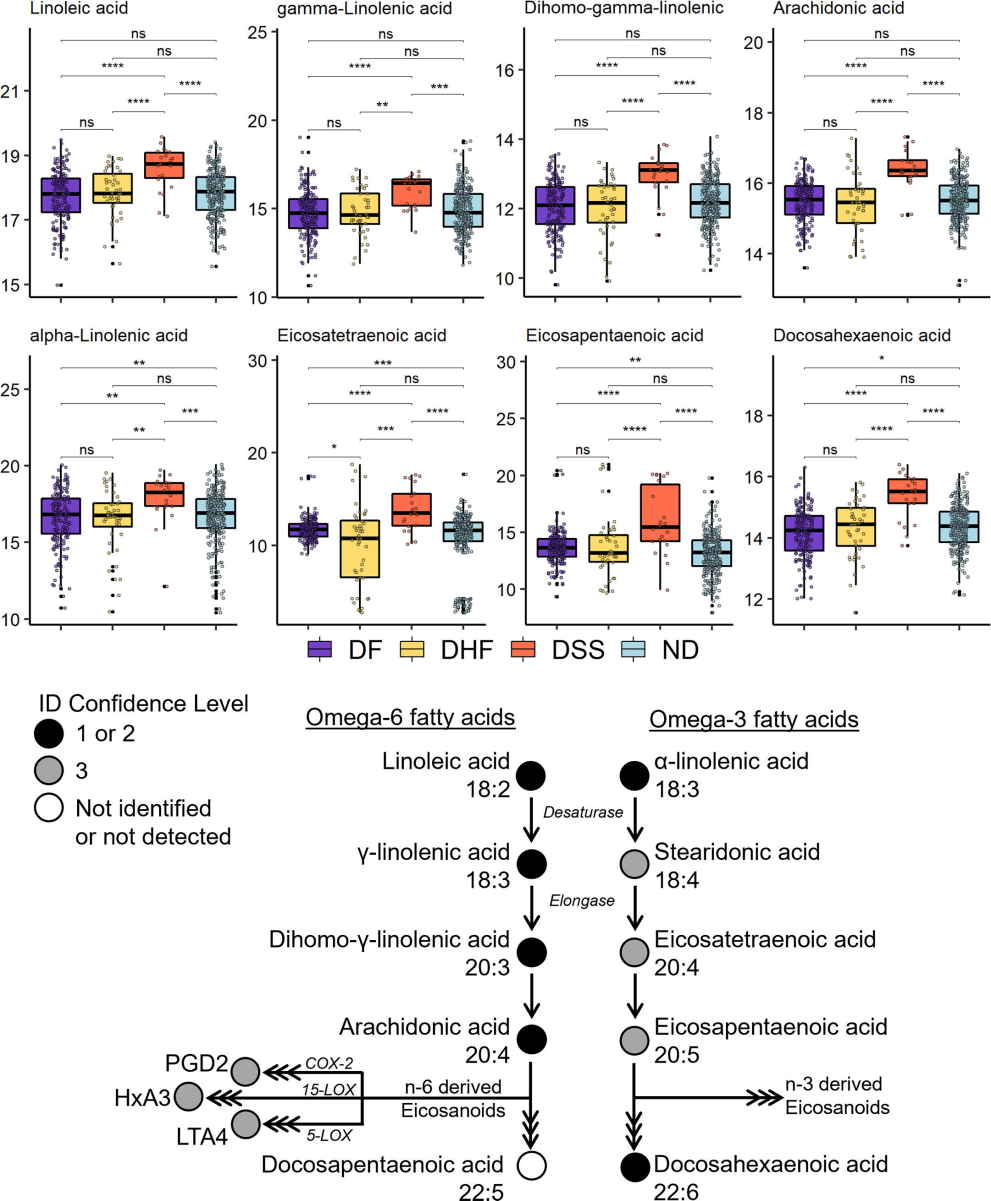
Fatty acid metabolism is perturbed in dengue disease. (Top) Abundance of n-6 and n-3 FAs in ND and dengue patients. Abundance is represented as the normalized and log2-transformed LC-MS peak area. Benjamini-Hochberg adjusted p-values generated using a moderated *t*-test were used to define statistical significance level; where ns is not significant and *P* < 0.0001, 0.001, 0.01, and 0.05 are each represented by ****, ***, **, and *, respectively. (Bottom) Fatty acids measured in patient samples. The color of each dot in the pathway corresponds to the identification confidence level, where 1 is the highest and 3 is tentative identification. Triple arrows between metabolites indicate that multiple metabolic steps are required.

The abundances of omega-3 (n-3) and omega-6 (n-6) fatty acids (FA) (Fig. 7; Table S4) and their downstream bioactive lipids were assessed (Fig. S6 and Table S4). Linoleic acid (18:2 n-6), a dietary FA, and three downstream FAs within the desaturation and elongation pathway were identified at confidence level 1 by comparison to synthetic standards. Three bioactive eicosanoids within different pathways of the n-6 arachidonic acid cascade were tentatively identified based on molecular formula prediction by accurate mass (confidence level 3). Regarding the n-3 FAs, α-linolenic acid and docosahexaenoic acid were identified. Three other n-3 FAs downstream of α-linolenic acid were tentatively identified based on molecular formula prediction by accurate mass. All the lipids assessed within n-6 and n-3 FA metabolism were observed to be more abundant in DSS patients compared to all other disease states.

### Bioactive sphingolipids are perturbed in dengue disease

Four bioactive sphingolipids were observed in the patient samples: sphingosine (d18:1), sphingosine-1-phosphate, sphinganine (d18:0), and sphinganine-1-phosphate (Fig. S6). Elevated levels of sphingosine (d18:1) and sphinganine (d18:0) were measured in DSS patients compared to all other disease conditions. Decreasing levels of sphingosine-1-phosphate and sphinganine-1-phosphate were measured in DHF and DSS patients in a disease severity-dependent manner.

### Other metabolites of interest

Related to purine metabolism, hypoxanthine and inosine were significantly lower in abundance in severe disease (Fig. S6). Amino acids, dipeptides, carnitines, and others are visualized in Fig. S7. Glycerophospholipids and glycerolipids are visualized in Fig. S8. All identified metabolites of interest are described in Table S2 and S3.

## DISCUSSION

### Circulating metabolic profile and biosignature development

To characterize the metabolic changes during dengue disease, circulating metabolites were measured using untargeted LC-MS/MS. Pathway analysis enabled the generation of hypotheses and metabolic pathways of interest and was supplemented by differential abundance analysis of individual features and metabolites. A disparity in the number of unperturbed features was observed between disease state comparisons. More unperturbed features for DF vs DHF than DF vs DSS indicated that more metabolic changes were measured in DSS. Regarding the direction of differential abundance, most features were less abundant in more severe forms of dengue disease, especially DSS. The direction of change in disease states may have implications as correlates of protection (higher in DF, lower in DSS) or as correlates of severe disease pathogenesis (lower in DF, higher in DSS). Thus, interesting features were structurally identified at confidence levels 1 or 2 for use as biomarkers and to enable inferences about their biological roles.

Multiple metabolomics studies involving DENV-infected patients have been conducted, all of which have contributed potential biomarkers from diverse sample backgrounds (10–14). Previous findings in adult patients corroborate many of the findings presented here in pediatric patients. Cui et al. observed reduction of dipeptides leucyl-alanine, phenylalanine, and serinyl-cysteine in adult DHF patients compared to DF (11–13). These studies also reported decreased serotonin and tryptophan metabolites in adults with DHF. Additionally, there is some agreement between the adult and pediatric data on the direction of lysophospholipid perturbation in DHF.

This current pediatric study contributes novel distinguishing biomarkers of pediatric severe dengue disease by leveraging a large dataset generated from 535 samples, including 22 DSS patients. Twenty-eight of the identified metabolites were used as a biomarker panel to classify disease state using the pediatric patient samples.

Additionally, we observed trends in this pediatric metabolomics data set that matched well-established trends in the medical field. For example, it is well known that creatinine levels increase as a function of age in children and adults (31), independent of sex. In our data set, the positive correlation between creatinine and patient age recapitulated these well-established trends (Fig. S4). Creatinine levels in serum or urine are commonly used as a measure of renal health. Kidneys filter creatinine out of the blood, such that an increase in serum creatinine levels may be a sign of liver dysfunction.

### Disease state classification

Classification success for DF vs DHF/DSS implies that phenotype differences were reflected in the patients’ circulating metabolome. Only one DHF/DSS sample was misclassified as DF in the full sample set analysis, and all samples were correctly classified by a subset analysis for days 3 to 6 of illness. Exclusion of plasma samples did not adversely affect classification performance. Furthermore, poor classification performance for ND vs DF was likely due to less phenotype difference between febrile DF and other febrile illnesses (ND).

Classification using the 28-metabolite biomarker panel revealed potential for prediction of severe dengue disease, as well as supported the hypothesis that the host metabolome contains dengue disease phenotype information. The biomarker panel presented here should be further validated in diverse sample sets representing different geographic locations, genetic backgrounds, and age ranges.

### Dipeptides

In this study, four identified dipeptides were reduced in DHF/DSS patients and were critical for disease state classification. Dipeptides have been used to treat human papillomavirus-associated disease (32), and a naturally occurring anti-HIV-1 dipeptide was discovered in elite controllers of HIV-1 (33, 34). Furthermore, dipeptidyl peptidase-4 (DPP4) inactivates bioactive peptides via cleavage of X-proline or X-alanine dipeptides from their N-terminus (35). DPP4 plays a role in immunity, cancer, and diabetes, but also antagonizes vasoconstriction and platelet aggregation via cleavage of NPY1-36, a neuropeptide that can be found in endothelial cells (36). It is intriguing that two of the highly perturbed dipeptides in DHF/DSS patients (prolyl-proline and leucyl-alanine) contain peptide motifs that are cleavable by DPP4. To our knowledge, this is the first evidence that exhibits association between dipeptides and dengue disease, and this association should be studied further.

### Tryptophan metabolism

Tryptophan can be metabolized into bioactive molecules that function in inflammation and aging, gut-brain homeostasis, immune regulation, cardiovascular diseases, and endothelial dysfunction (37–41). Tryptophan metabolism is also implicated in infectious diseases, including DENV infection and disease (10–12). Importantly, dysregulation of tryptophan metabolism observed within our data set is consistent with, and significantly expands upon, the knowledge gained from previous findings (12).

Hydroxylation of tryptophan via tryptophan hydroxylase (TPH) leads to the formation of bioactive serotonin. Serotonin and its precursor, 5-hydroxytryptophan, were identified in this study. 5-Hydroxytryptophan is the direct precursor of serotonin. The elevated 5-hydroxytryptophan levels in DF and DHF compared to ND patients could be a protective metabolic response, in which host metabolism shifts to replenish serotonin levels. In DSS patients, 5-hydroxytryptophan levels are not significantly different from ND patients and are significantly lower than DF patients. The lack of elevated 5-hydroxytryptophan levels in DSS may indicate that patient metabolism did not effectively shift to supplement depleted serotonin levels. It is also important to note that 5-hydroxytryptophan did not show the same decrease in DSS that was observed for serotonin. Therefore, as hypothesized in a previous study (12), normal 5-hydroxytryptophan levels in DSS patients indicate that serotonin synthesis is not the major perturbation; rather, serotonin release or uptake is highly perturbed.

Serotonin abundance was highest in ND patients, progressively lower in DF and DHF, and drastically depleted in DSS patients. Platelets trended similarly to serotonin, except that they were not significantly depleted in DSS compared to DHF patients. Additionally, serotonin in DF and DHF patients trended with day of illness (Fig. S5), resembling previously reported platelet count behavior during DF progression, where platelets hit a low on day 6 and increased to normal by day 10 (42). A similar platelet trend for days 1 to 6 was observed in this study. Thus, it is hypothesized that serotonin abundance may also recover by day 10 in DF patients. These findings contribute to the hypothesized connection between platelets and serotonin but suggest diverse roles for serotonin, indicating that mechanisms other than thrombocytopenia may contribute to the depletion of circulating serotonin in DSS.

In the mediation of shock and vascular endothelial dysfunction, serotonin may either be protective or pathogenic. In a pathogenic role, serotonin has been shown to induce local vasodilation when released at the vascular endothelium, affecting endothelial function and contributing to vascular leakage and shock (43, 44). Systemic shock can be induced by circulating antibody-antigen immune complexes via the platelet Fcγ receptor IIA (44). Within this immune complex-induced mechanism, serotonin is pathogenic and is required for vasodilation. Serotonin can also be derived from mast cells and induce significant platelet activation, leading to thrombocytopenia via platelet aggregation and increased splenic uptake (45). However, platelet activation is also necessary for hemostasis and vascular wall maintenance. Thus, serotonin may also play a protective role.

The reason for circulating serotonin depletion in dengue disease is still unclear. One potential serotonin sink in dengue disease could be the liver. Liver damage is a clinical presentation of severe dengue disease, indicated by increased levels of liver transaminases and bilirubin in circulation. Multiple studies involving DENV-infected adults and children reported that greater than 90% of patients had elevated circulating liver aminotransferases (46). Through altered expression of serotonin receptor subtypes in the liver (e.g., 5-HT_2A_ and 5-HT_2B_ serotonin receptors), serotonin mediates hepatocyte proliferation and restoration of hepatic mass following injury (47–50). Therefore, the uptake and usage of serotonin by hepatocytes to counteract DENV-induced liver damage may, in part, account for decreased circulating serotonin levels.

Decreased production of albumin by the liver (e.g., liver damage) or increased escape of circulating albumin from the vascular space (increased vascular permeability) can lead to hypoalbuminemia (51). Two studies involving DENV-infected children reported that 60–80% of children present with hypoalbuminemia (46). In blood, most tryptophan (90%) is bound to albumin, and 10% of tryptophan is unbound (free) and available for tissue uptake. Therefore, decreased tryptophan levels detected in dengue patients may be related to decreased albumin, which was supported by the positive correlation observed between the two measures. Also, rapid equilibration between albumin-bound and free tryptophan in blood, paired with sustained tissue uptake, results in depleted tryptophan blood levels (52). Tissue uptake and subsequent usage of tryptophan through one of its four metabolic pathways, without sufficient replenishment of tryptophan, could be another reason for decreased circulating tryptophan in dengue disease.

IPA has been reported to have immunomodulatory, anti-inflammatory, and antioxidant effects, as well as various protective functions in mammals (39, 53). IPA abundance has a positive correlation with factors that promote cardiovascular health, and IPA levels decrease in scenarios where cardiovascular health is diminished (54). Decreased IPA levels in DHF and DSS patients could be related to diminished cardiovascular health. Myocarditis has been reported but is not common in dengue disease.

### Fatty acid metabolism

Lipid metabolism is dysregulated in all disease states in our data set; specifically, FA metabolism and biosynthesis. FAs and other cellular lipids are important structural, signaling, and energy-yielding molecules for viral entry, replication, assembly, and release. Previous work has shown that FA biosynthesis is actively dysregulated during viral infection to benefit assembly and function of viral replication factories (20, 55–57). FA oxidation pathways and the citric acid cycle (TCA) were observed to be altered across disease states. DENV utilizes FA beta-oxidation to fuel the mitochondrial TCA cycle, which in turn provides adenosine-5′-triphosphate (ATP) as energy for the viral lifecycle. Cellular lipids can also mediate the inflammatory response associated with disease. Increased linolenic acid (n-3) and linoleic acid (n-6, precursor to arachidonic acid) metabolism leads to formation of the pro- and anti-inflammatory and bioactive lipid mediators, such as eicosanoids (leukotrienes, prostaglandins, thromboxanes) (58–60).

Linoleic acid (18:2 n-6) is an essential n-6 FA that is metabolized to arachidonic acid or other very-long-chain FAs. Enhanced levels of linoleic acid and metabolites serve to increase pools of arachidonic acid and its downstream bioactive lipids. Three eicosanoids were tentatively identified in this study and were elevated in DSS patients (Fig. S6): leukotriene A_4_ (LTA_4_), prostaglandin E_2_ (PGE_2_), and hepoxilin A_3_ (HxA_3_). These three molecules may have implications for increased vascular permeability observed in severe dengue disease.

Leukotrienes are produced by the enzyme 5-lipoxygenase (5-LOX). Initially, 5-LOX generates 5-hydroperoxyeicosatatraenoic acid, which is unstable and rapidly converted to 5-hydroxyeicosatetraenoic acid or to LTA_4_, which is then converted to LTB_4_ or LTC_4_. LTC_4_ serves to increase vascular permeability and plasma leakage (61, 62), both of which are symptoms observed in DHF and DSS.

Prostaglandins are produced via arachidonic acid through cyclooxygenase (COX) isoenzymes. PGE_2_ is significantly increased during inflammation, where it causes increased microvascular permeability (59, 63). Another study demonstrated involvement of COX-2 in DENV replication in cell culture (64), potentially implying a virally induced mechanism for increased PGE_2_. HxA_3_ is a non-canonical eicosanoid that is produced when arachidonic acid is converted via the 12S-LOX enzyme to 12SHpETE, the HxA_3_ precursor. HxA_3_ increases vascular permeability in rat skin, induces neutrophil chemotaxis, and stimulates release of arachidonic acid and diacylglycerol (65, 66). Elevated circulating arachidonic acid levels could, in part, be related to HxA_3_ activity.

### Sphingolipids

Sphingosine is the common backbone of the diverse sphingolipid molecules, which can have signaling and structural roles. Sphingosine, or the closely related sphinganine, can be phosphorylated to generate a potent signaling molecule. Based on ELISA measurements of adult dengue patient sera from Colombo, Sri Lanka, sphingosine-1-phosphate decreased in a severity-dependent manner when compared to healthy controls (67). This current mass spectrometry-based study of pediatric patients from Nicaragua recapitulated the disease severity-dependent decrease in sphingosine-1-phosphate and revealed that this trend extends to DSS patients. Like serotonin, sphingosine-1-phosphate is stored in and released by platelets (68). Thus, these trends may be associated with thrombocytopenia.

Sphingosine-1-phosphate and its various receptors (S1PR1 through S1PR5) have been implicated in both protection and disruption of the endothelial barrier. Upon binding of sphingosine-1-phosphate, S1PR1 promotes regulation of endothelial cell function via downstream signaling. In contrast, S1PR2 induces vascular permeability via the Rho-ROCK-PTEN signaling cascade that facilitates phosphorylation and loss of VE-Cadherin at adherens junctions (69–71). Modak et al. hypothesized that low serum sphingosine-1-phosphate and DENV-induced upregulation of the high-affinity S1PR2 in endothelial cells result in preferential activation of disruptive signaling pathways leading to vascular permeability (71).

Sphinganine-1-phosphate was progressively decreased in DF, DHF, and DSS patients in this study (Fig. S6). Sphinganine-1-phosphate lacks a double bond and is thus closely related to sphingosine-1-phosphate and can bind S1P receptors. Sphinganine-1-phosphate was reportedly depleted in mice after hepatic ischemia-reperfusion, and exogenous replenishment of sphinganine-1-phosphate protected the mice against liver and kidney injury, improving endothelial integrity and vascular function (72). The protective effect of sphinganine-1-phosphate on endothelial cells was found to be related to S1PR1 (73). Thus, depleted sphinganine-1-phosphate in DENV-infected patients may be related to vascular integrity.

### Inosine and hypoxanthine

Decreased levels of inosine and its downstream product hypoxanthine (Fig. S6). In dengue disease, especially severe disease, could be related to higher levels of adenosine (inosine precursor) and lower levels of xanthine and uric acid (hypoxanthine catabolites). Adenosine is a vasodilator and platelet aggregation inhibitor and thus could contribute to leak (74–77). Circulating adenosine was reported to inhibit polymorphonuclear leukocyte function, resulting in decreased synthesis of specialized pro-resolving lipid mediators during coagulation that drive resolution of inflammation (78). Additionally, circulating adenosine deaminase abundance and activity (the enzyme that converts adenosine to inosine) are reportedly altered in various diseases (79, 80). Adenosine was not detected in this data set, but its potential importance warrants further investigation.

### Study limitations

Biomarkers with potential for triaging severe disease should be perturbed and measurable within the first three days of the acute phase of dengue disease. This study included samples that were collected from day 0 to 6 of illness. Thus, the perturbed metabolites described here were associated with DHF/DSS but were not used to predict progression to DHF/DSS. The next step towards identifying biomarkers for early triage will require analyzing the biomarkers presented here in samples from patients on day 3 of illness or earlier who later progress to severe disease. Additionally, further expansion of these studies to other geographical regions, genetic backgrounds, age ranges, and longitudinal sample collections will help to fortify such biomarker panels.

This study did not include healthy controls. Instead, we used the ND control and various dengue disease severities to assess the circulating metabolic changes that were specific to DF febrile illness and metabolic changes between DF and DHF/DSS.

This study included majority serum and some plasma samples, but disease state classification was equally successful with or without inclusion of plasma samples in the training and test sets. Serum sample preparation involves platelet coagulation, which should be considered when interpreting the relationship between platelets and serum metabolites. Additionally, coagulation can induce production of arachidonic acid-derived lipid mediators.

Like all metabolomics studies, this study had an inherent measurement bias based on molecular polarity. The extraction protocol and LC-MS/MS analyses were optimized for mid-polar molecules, so additional workflows would be necessary if comprehensive analyses of highly polar metabolites, sugars, or nonpolar lipids are desired.

### Conclusion

Here, we demonstrate that the pediatric circulating metabolome is dynamically altered in response to DENV infection and reveals signatures associated with disease severity. Dengue disease state was accurately classified, and the biochemistry of severe dengue disease pathogenesis was explored. Unique to this study was the identification of the metabolic biosignatures of DSS, and biomarkers of DHF were identified that were either novel or that recapitulated those reported in studies from other geographical regions, supporting the hypothesis that the metabolome can provide information that is independent of age, and geographical and genetic backgrounds. These studies could be critical in assembling biomarkers for early triaging of severe dengue disease.

## MATERIALS AND METHODS

### Chemicals and reagents

LC-MS grade water and methanol were purchased from Honeywell (Charlotte, NC). LCMS grade acetonitrile was purchased from Fischer Scientific (Hampton, NH). Analytical grade 5-hydroxy-L-tryptophan, arachidonic acid, arachidonic acid-d_8_, creatinine, creatinine-d_3_, dihomo-gamma-linolenic acid, dihomo-gamma-linolenic acid-d_6_, gamma-linolenic acid, indole-3-acetic acid, indole-3-lactic acid, indole-3-propionic acid, linoleic acid, linoleic acid-d_4_, L-kynurenine, L-tryptophan, serotonin hydrochloride, and serotonin-d_4_ hydrochloride were purchased from Cayman Chemical (Ann Arbor, MI). Analytical grade carnitine, hypoxanthine, and inosine were purchased from Sigma Aldrich (St. Louis, MO).

### Study design

For this study, 535 samples from 535 individuals were retrospectively obtained from two different well-established studies in Managua, Nicaragua (81, 82). A set of 122 well-characterized samples collected between 2012 and 2013 was sourced from the Pediatric Dengue Cohort Study (PDCS), following over 3,800 children between the ages of 2 and 17 years old since 2004 (Fig. 1). Cohort patients were enrolled as healthy volunteers, followed for all medical episodes, and monitored for suspected arboviral diseases. Those who met the case definition of dengue, or undifferentiated febrile illnesses, were worked up for laboratory confirmation using molecular biological, virological, and/or serological methods. Another 413 samples were obtained from patients aged 1 to 14 years old (Fig. 1) in the Pediatric Dengue Hospital-based Study (PDHS), who presented at the Hospital Infantil Manuel de Jesús Rivera, the National Pediatric Reference Hospital in Nicaragua, between 2005 and 2015, with a fever or history of fever for <7 days and one or more of the following signs and symptoms: headache, arthralgia, myalgia, retro-orbital pain, positive tourniquet test, petechiae, or signs of bleeding. Cases were laboratory-confirmed for DENV infection by detection of DENV RNA by RT-PCR, isolation of DENV, seroconversion of DENV-specific IgM antibody titers observed by MAC-ELISA in paired acute- and convalescent-phase samples, and/or seroconversion or a ≥4-fold increase in anti-DENV antibody titer measured using inhibition ELISA (iELISA) in paired acute and convalescent samples (83–85). Immune status was determined using iELISA in early convalescent samples (14 or more days post-onset of symptoms); a titer <2,560 was considered primary infection, and ≥2,560 was considered secondary infection. Cases were classified by disease severity (DF, DHF, or DSS) using computerized algorithms based on the 1997 WHO schema (21). Samples that were negative for DENV infection were classified as ND. Metadata recorded included patient sex, age, disease outcome (disease state), infection history, DENV serotype, day of illness (number of days of fever at time of enrollment and sample collection), date of sample collection, and detailed clinical data across disease evolution.

These samples were procured as part of the normal dengue diagnosis mission of the laboratory and not as part of an experimental protocol. Parents or legal guardians of participants provided written informed consent; participants 6 years of age and older provided assent; and participants aged 12 years and older in the Hospital-based Dengue Study provided written assent.

### Sample preparation and metabolite extraction

Twenty microliters of each serum sample were aliquoted into individual microcentrifuge tubes, and an additional 20 µL of each serum sample were combined to generate the pooled quality control (QC). For metabolite extraction, patient serum and pooled QC samples were randomized. Five microliters of L-tryptophan-d_5_ (80 ng/mL) heavy-isotope=labeled internal standard were added to 20 µL of patient serum (or pooled QC aliquot) in a microcentrifuge tube. To precipitate proteins, 100 µL of cold methanol was added to the serum, and samples were incubated for 12 h at −80°C. Samples were then centrifuged at 4°C for 15 min at 18,000 × *g* to pellet proteins. Supernatant was then transferred to a new microfuge tube and dried under nitrogen. Samples were reconstituted in 25 µL of methanol/water (50/50), allowed to stand at room temperature for 15 min, vortexed for 20 sec, and centrifuged to pellet insoluble debris. Sample supernatants were then transferred to autosampler vials fitted with low-volume inserts and immediately submitted for LC-MS analysis. Serum samples were prepared and analyzed in six randomized batches. A pooled quality control sample was run after every five experimental samples, and a solvent blank was run after every 10 samples.

### Untargeted liquid chromatography-mass spectrometry

Sample order for injection was randomized. Each sample was injected (7.5 µL) with an Agilent 1290 HPLC system where metabolites were separated on an XBridge BEH C18 column (2.5 µm particle size, 2.1 × 100 mm, Waters Millford, MA, USA). The total mobile phase flow rate was 0.250 mL/min, made up of water + 0.1% formic acid (mobile phase A) and 95/5 acetonitrile/water + 0.1% formic acid (mobile phase B). For metabolite separation, the mobile phase composition began at 5% B and held until 0.5 min. From 0.5 to 14 min, the mobile phase composition was adjusted in a linear fashion to 98% B. From 14.5 to 15 min, the mobile phase composition was returned to starting gradient conditions of 5% B. From 15 to 19.5 min, the starting gradient conditions were held to equilibrate the LC column for the subsequent sample injection. The LC column outlet was coupled to the electrospray ionization (ESI) source of an Agilent 6224 time-of-flight mass spectrometry system being operated in positive ionization mode. The ESI emitter was electrically grounded, and the MS-inlet capillary was held at −4,000 V to generate and transmit positive ions from the metabolites eluting from the LC column. The ESI nebulizer nitrogen gas was set to 45 psi, and the heated counter flow of nitrogen (“dry gas” used to aid in droplet desolvation) was flowed at 10 L per minute and held at 310°C. Beyond the MS-inlet capillary, the fragmentor voltage was set to 120 V to aid in ion desolvation and transmission, and the skimmer voltage was set to 50 V. The peak-to-peak voltage of the ion transfer octopole was set to 750 V. The time-of-flight mass analyzer was set to scan between *m/z* 70 to 1,700, collecting full-scan (MS1) spectra at a rate of 1.66 spectra/sec.

### Metabolite identification via liquid chromatography-tandem mass spectrometry

To confirm metabolite identity, RT, *m/z*, and the collision-induced dissociation (CID) product ion spectra were collected for each metabolite. For level 1 identification, RT, *m/z,* and CID product ion spectrum from a pure synthetic standard were matched to that of the molecule originating from serum. For level 2 identification, RT, *m/z,* and CID product ion spectra for the molecule in serum were compared to literature, spectral databases, or known dissociation patterns (86–88). For level 3 identification, the accurate mass (m/z) of a feature measured by the time-of-flight (TOF) mass analyzer was tentatively assigned a molecular formula (within 20 ppm mass error to the exact mass) and structure. Confirmation of metabolite identities was performed on an Agilent 1290 HPLC system coupled to an Agilent 6546 quadrupole time-of-flight (QTOF) mass spectrometry system. LC conditions, ionization polarity, and ion transfer optics in the MS were identical to conditions used for the untargeted LC-MS experiment. To acquire CID product ion spectra for metabolites of interest, the quadrupole mass analyzer was set to transmit the precursor *m/z* of interest with an isolation width of 1.3 Da. Precursor ions were transmitted through the quadrupole mass analyzer and accelerated into the collision cell filled with N_2_ gas (24 psi). Within the collision cell, precursor ions underwent energetic collisions with N_2_ gas molecules. Product ion spectra were collected at collision energies of 10 and 40 arbitrary units. After the collision cell, precursor and product ions were pulsed into the TOF for mass analysis and subsequent detection.

### Data processing

#### Molecular feature extraction

The Agilent data files were converted from .d to .mzML format using ProteoWizard MS Convert version 3.0.6478 64 bit. Peak picking, retention time correction, chromatogram alignment, and gap filling were performed using XCMS software version 1.46 in R version 3.2.2 (89). The R package IPO was used to optimize XCMS parameters on the dataset (90), and CAMERA was used for deisotoping (91).

#### Molecular feature description

Within each data file, the abundance and tentative identity of many potential metabolites (molecular features) are embedded. Two LC-MS metrics that define a molecular feature are the RT of its chromatographic peak and the *m/z* of the ion that correlates to the chromatographic peak. Enabled by the accurate mass (*m/z*) measurement capability of the time-of-flight mass analyzer, each feature was tentatively identified by searching its measured accurate mass (within ± 20 parts-per-million mass error) against the Human Metabolome Database (HMDB), LipidMaps, Metlin, and Kegg databases using CEU mass mediator (92). Additionally, the area under the chromatographic peak of a molecular feature represents its abundance in each serum sample.

#### Preprocessing

All data preprocessing steps were conducted in R version 3.4.2 (93). Features were first filtered to remove any that failed to meet the following criteria: (1) present in at least 20% of all samples across all analysis batches; (2) present in at least 75% of all pooled QC samples; and (3) present in at least 70% of samples from at least one disease group (ND, DF, DHF, DSS).

Normalization was conducted stepwise. First, within each batch separately, features that were not present in at least 80% of pooled QC samples in that batch were removed. Features were then normalized using a Tobit regression (left-censored at the minimum value for each batch) fitted to the pooled QC samples, implemented in the R package “AER” version 1.2.5 (94, 95). Batches were then combined, retaining only those features present in all batches, and normalized feature-wise across all batches by the ratio of pooled QC mean batch abundance to pooled QC overall mean abundance.

The year of sample collection influenced the presence or abundance of some features, suggesting that length of storage and/or methods of collection and handling may be influencing the results. Therefore, features were removed if found to be present in ≥50% of all samples (irrespective of group) only in samples collected between 2005 and 2009, or only in samples collected between 2011 and 2015. However, if the feature appeared to be specific to a sample infected with a single serotype (i.e., found in >50% of samples of that serotype and <50% of samples from other serotype), then the feature was retained. Finally, features that had a coefficient of variation >30% in pooled QC samples after combining and normalizing batches were deemed unreliable and removed. Abundances were log_2_-transformed. Missing values were imputed using a random forest algorithm implemented in the R package missForest version 1.4 (96). Abundance variance was calculated for each feature across all samples, and features in the lowest quartile were excluded from analysis (97).

### Metabolic pathway analysis

Differential abundance and statistics (t-score and p-value) were calculated feature-wise via *limma* for each pairwise comparison of dengue disease severity. Pathway analysis was performed on results from each pairwise disease state comparison. Feature *m/z*, RT, p-value, and t-score were used as inputs for the *mummichog* metabolic pathway analysis algorithm (98). For *mummichog* version 2.0 analysis, primary ion types were forced, the metabolite p-value cutoff was 0.05, a minimum of three metabolites were required to flag a pathway, and the pathway library was *Homo sapiens* (MFN). A 20 ppm mass error tolerance was set for tentative metabolite annotation.

### Statistical analysis

Univariate analysis of features was implemented in the R package *limma* version 3.32.10, in which linear models with empirical Bayes statistics were applied feature-wise to generate pairwise comparison of feature abundances across disease states (99–101). The models provided estimated log2 FCs, moderated t-statistics, and false discovery rate adjusted p-values for each feature. Significant features were defined by log_2_FC ≥ 1 and *P*-value < 0.05 after adjustment for false discovery rate via the Benjamini-Hochberg method.

To develop classification models, the 535 samples were randomly divided within disease states into a training set (75% of samples within each disease state) and a test set (remaining 25% of samples), where disease states were ND, DF and severe disease (DHF/DSS). DHF and DSS were combined into the severe disease category to alleviate the small sample size for each disease state. Training sets were used to fit models. Test sets were used only to test predictions from fitted models.

Using the training sample set, an ensemble of classification models was constructed for two separate classification problems: ND vs DF and DF vs DHF/DSS (102–104). The R “caret” package version 6.0.93 was used to build several classification models (105), including a generalized linear model, random forest, adaBoost.M1, support vector machines, linear and quadratic discriminate analysis, and k-nearest neighbors. Parameters were chosen by five-fold cross-validation. Peak areas were scaled and centered. The generalized linear model, random forest, and AdaBoost.M1 gave perfect classification on the training set and were therefore chosen for evaluation with the test set.

## Data Availability

Data were uploaded to the Metabolomics Workbench study ST003699 (http://dx.doi.org/10.21228/M8V83J).

## SUPPLEMENTAL MATERIAL

Supplemental figures jvi.01572-25-s0001.docx Figures S1 to S8.

Supplemental tables jvi.01572-25-s0002.xlsx Tables S1 to S4.

ASM does not own the copyrights to Supplemental Material that may be linked to, or accessed through, an article. The authors have granted ASM a non-exclusive, world-wide license to publish the Supplemental Material files. Please contact the corresponding author directly for reuse.
