# Supplementary material for: Perturbed pediatric circulating metabolome in mild and severe dengue disease
Source: J Virol. 2025 Oct 29;99(11):e01572-25. doi: 10.1128/jvi.01572-25 (PMC12645983; doi:10.1128/jvi.01572-25)
Supplement: Supplemental figures — Figures S1 to S8. [file jvi.01572-25-s0001.docx]

**Supplementary Figures: Perturbed pediatric serum metabolome in mild and severe dengue disease**

**AUTHORS**

Paul S. Soma^1^, Rebekah C. Gullberg^1^, Barbara Graham^1^, M. Nurul Islam^1^, Angel Balmaseda^2,3^, Carol D. Blair^1^, Barry J. Beaty^1^, John T. Belisle^1^, Eva Harris^4^ and Rushika Perera^1^

**1** Department of Microbiology, Immunology, and Pathology, Colorado State University, Fort Collins, Colorado, United States of America, **2** Sustainable Sciences Institute, Managua, Nicaragua, **3** Laboratorio Nacional de Virología, Centro Nacional de Diagnóstico y Referencia, Ministry of Health, Managua, Nicaragua, **4** Division of Infectious Diseases and Vaccinology, School of Public Health, University of California, Berkeley, Berkeley, California, United States of America

**CORRESPONDING AUTHOR:** Rushika Perera; rushika.perera@colostate.edu

**LIST OF SUPPLEMENTARY FIGURES**

Figure S1a-b. Volcano plots displaying metabolite differential abundance between disease states

Figure S2. Frequency distribution of log_2_FC values for all LC-MS features

Figure S3a-n. LC-MS/MS validation of metabolite identities at confidence level 1

Figure S4. Creatinine abundance correlations with patient age

Figure S5. Serotonin and platelet trends as a function of patient days of illness

Figure S6. Metabolite abundance boxplots for eicosanoids, purines and sphingolipids

Figure S7. Metabolite abundance boxplots for amino acids, dipeptides, carnitines and other metabolites

Figure S8. Metabolite abundance boxplots for glycerophospholipids and glycerolipids

**
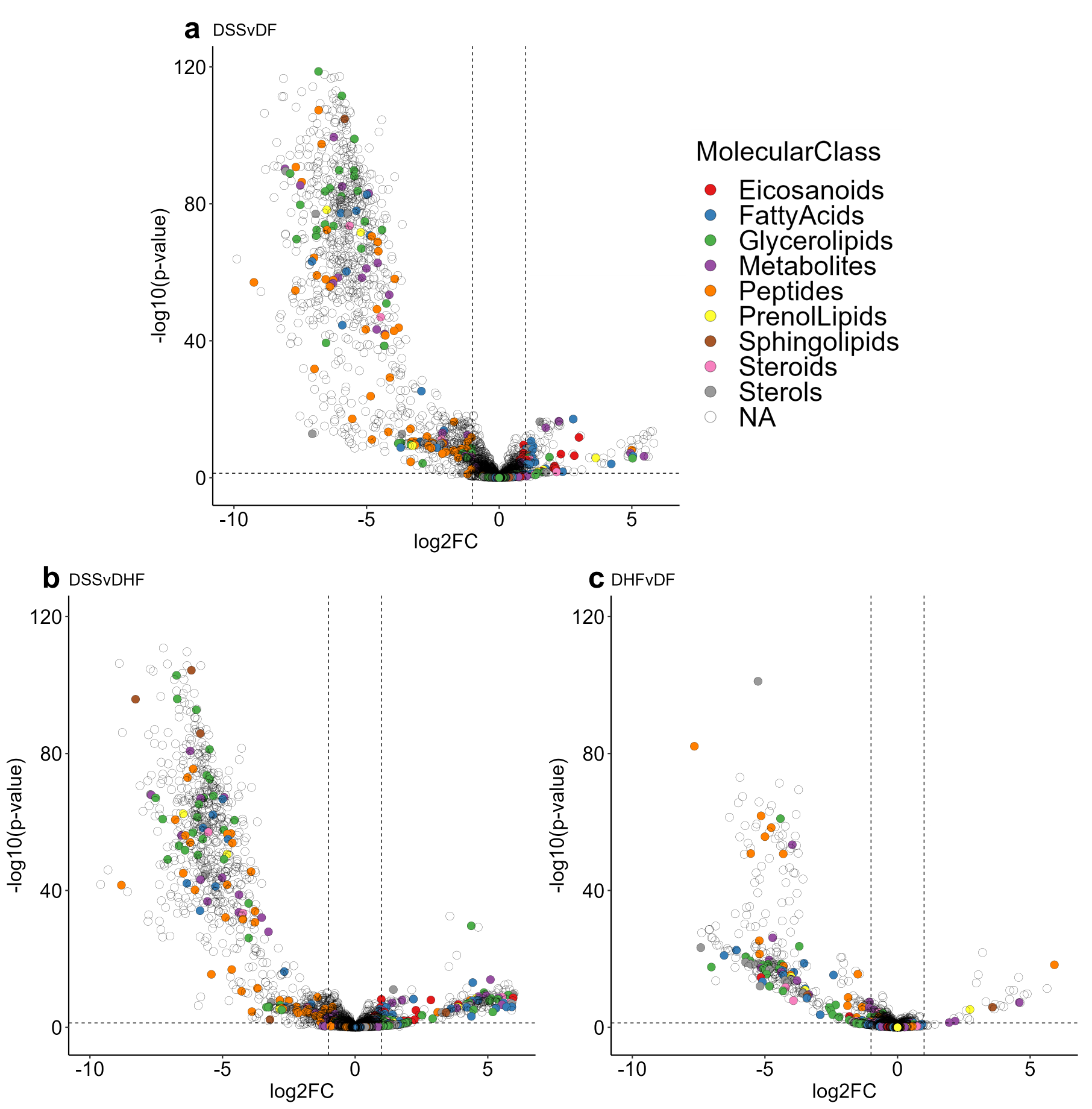
**

**Figure S1. Differential abundance of features between dengue disease states.** (**a**) Negative log_2_FC values can be interpreted as either a lower feature abundance in DSS or greater abundance in DF. Positive log_2_FC values can be interpreted as either a greater feature abundance in DSS or lower abundance in DF. (**b**) Negative log_2_FC values can be interpreted as either a lower feature abundance in DSS or a greater abundance in DHF. Positive log_2_FC values can be interpreted as either a greater feature abundance in DSS or a lower abundance in DHF. (**c**) Negative log_2_FC values can be interpreted as either a lower feature abundance in DHF or a greater abundance in DF. Positive log_2_FC values can be interpreted as either a greater feature abundance in DHF or a lower abundance in DF. Colored points represent a manually curated list of 266 tentatively identified (based on accurate mass) potentially biologically relevant molecules, where color represents the tentative molecular class. Noncolored points represent features that were either manually defined as not biologically relevant, or tentative identification based on accurate mass was unsuccessful. The horizontal dotted line represents an adjusted p-value of 0.05 and the vertical dotted lines represent log_2_FC = ± 1.

**
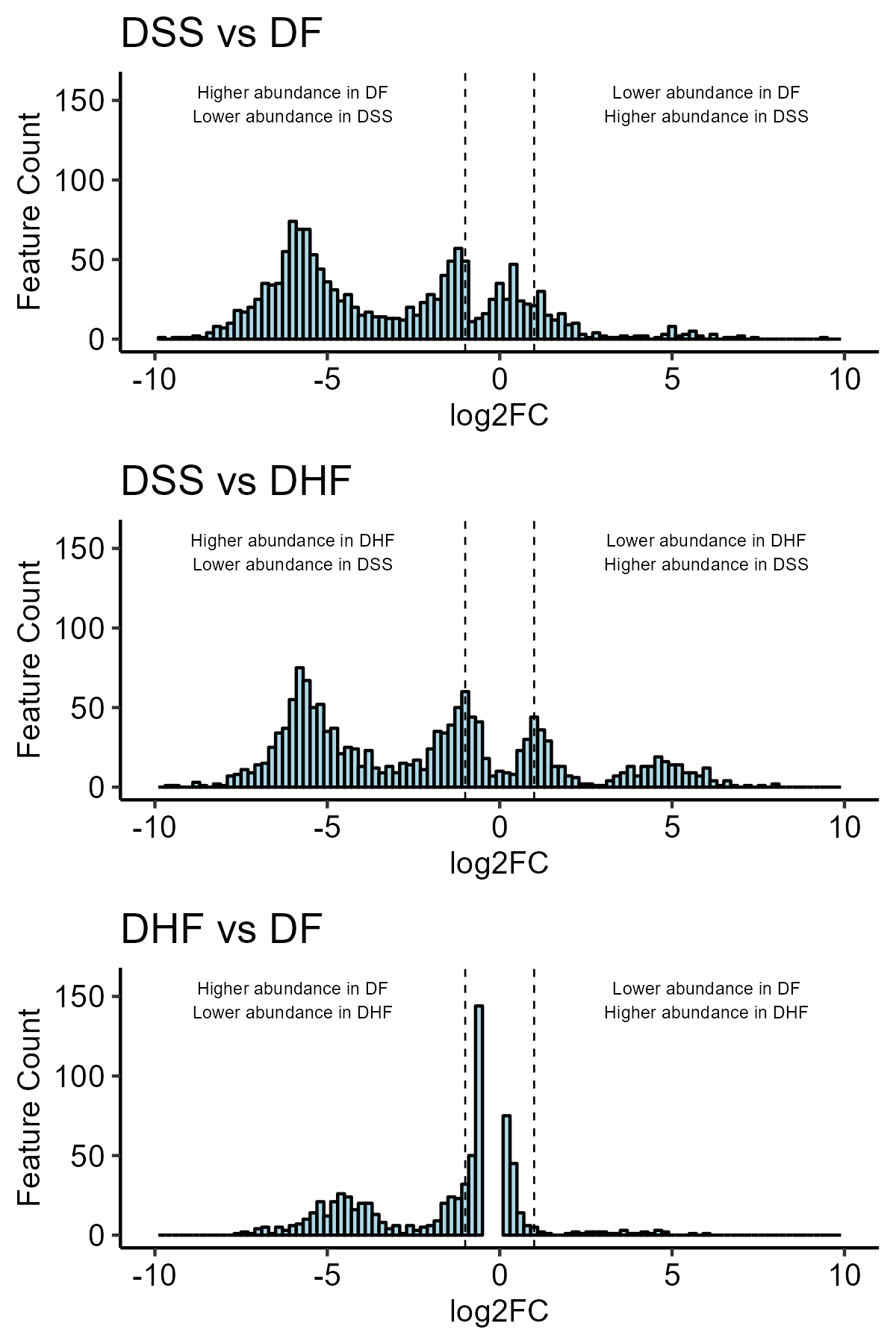
**

**Figure S2. Number of significantly perturbed features for each pairwise disease state comparison.** Frequency distribution of log2 FCs for the 1512 features that had significant differential abundance (adjusted p-value < 0.05) for three pairwise comparisons (top: DSS vs DF, middle: DSS vs DHF, bottom: DHF vs DF). Log_2_FC bin width was set to 0.2. Vertical lines represent log_2_FC = ± 1. Log2 FCs were calculated with the R package *limma.*


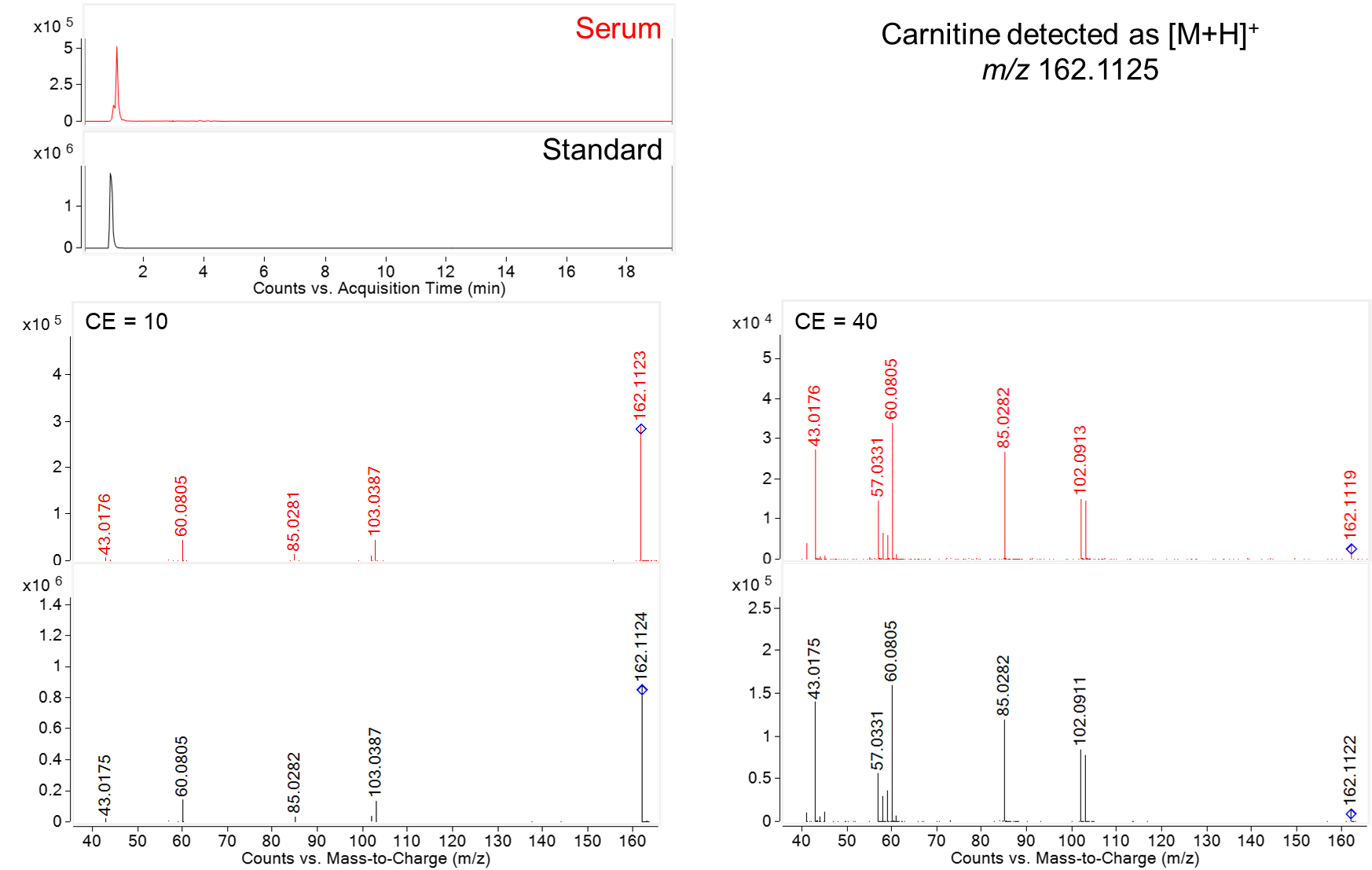
**Figure S3a**. **Carnitine detected as [M+H]^+^ at *m/z* 162.1125.** **Top**) Chromatographic retention time was matched between synthetic standard and the endogenous molecule detected in serum. **Bottom**) Collision-induced dissociation product ion spectra comparison for synthetic and endogenous molecules at collision energy values of 10 and 40.

**
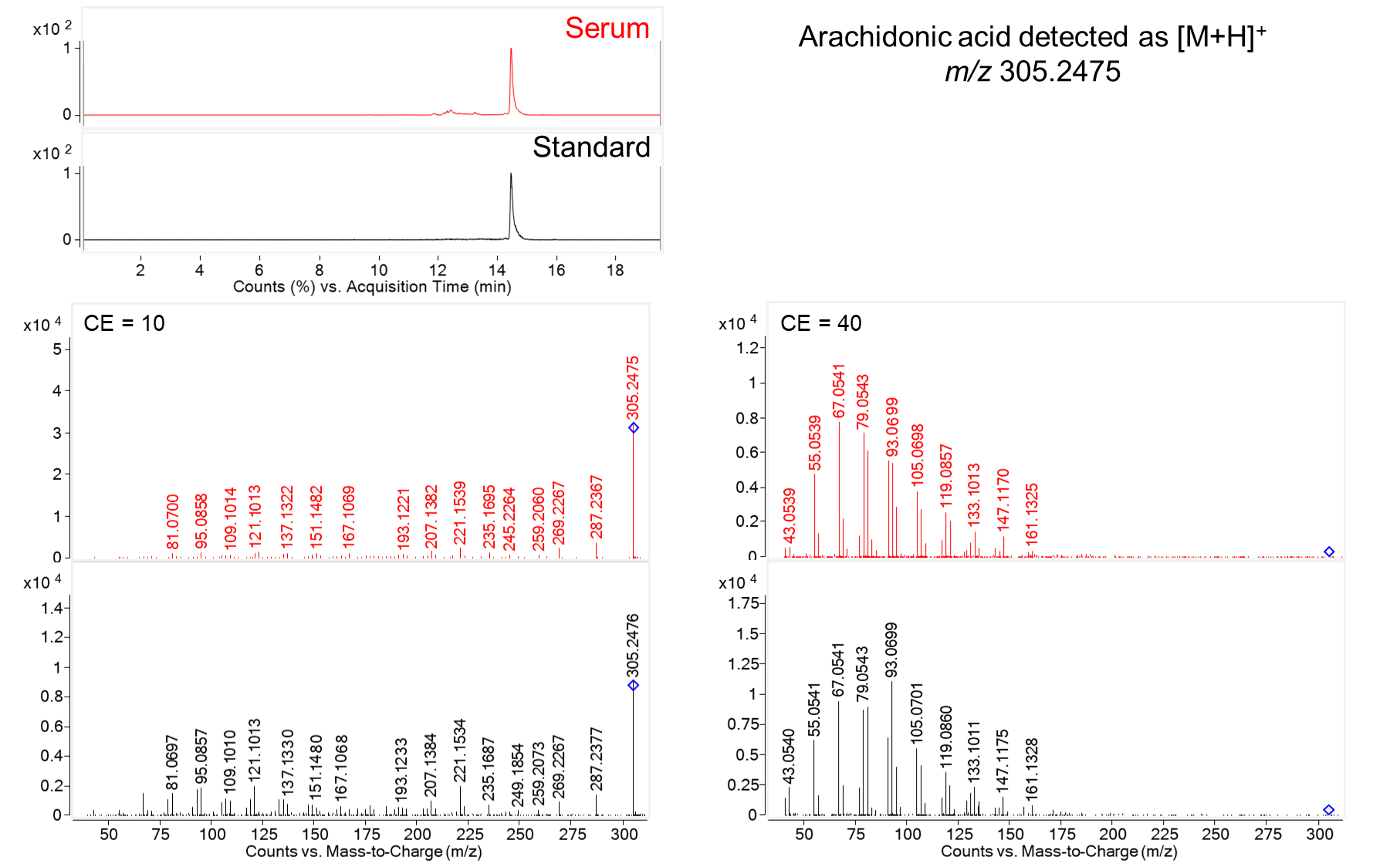
Figure S3b**. **Arachidonic acid detected as [M+H]^+^ at *m/z* 305.2475**. **Top**) Chromatographic RT was matched between synthetic standard and the endogenous molecule detected in serum. **Bottom**) Collision-induced dissociation product ion spectra for synthetic and endogenous molecules at collision energy values of 10 and 40.

**
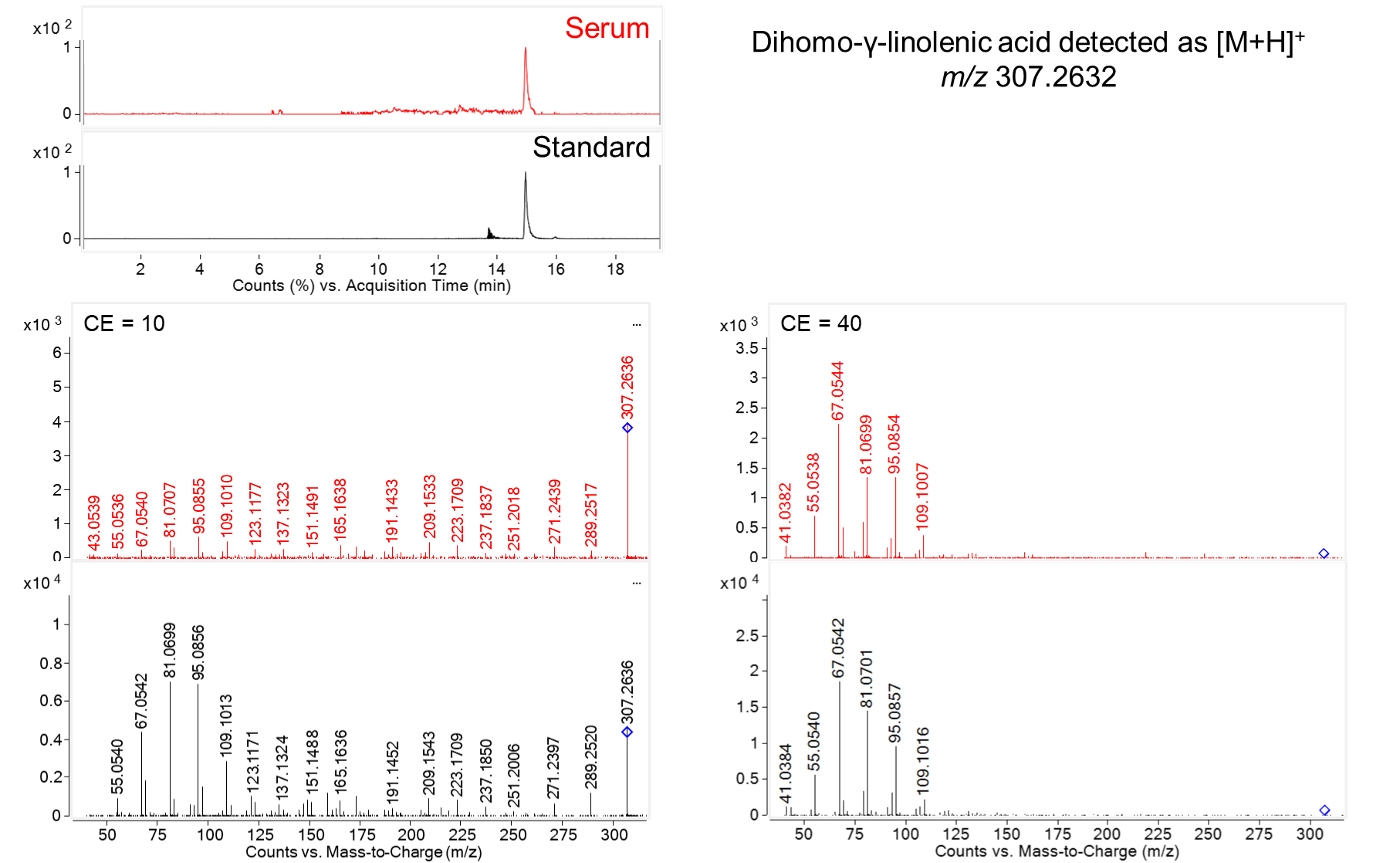
Figure S3c**. **Dihomo-γ-linolenic acid detected as [M+H]^+^ at *m/z* 307.2632.** **Top**) Chromatographic retention time was matched between synthetic standard and the endogenous molecule detected in serum. **Bottom**) Collision-induced dissociation product ion spectra comparison for synthetic and endogenous molecules at collision energy values of 10 and 40.

**
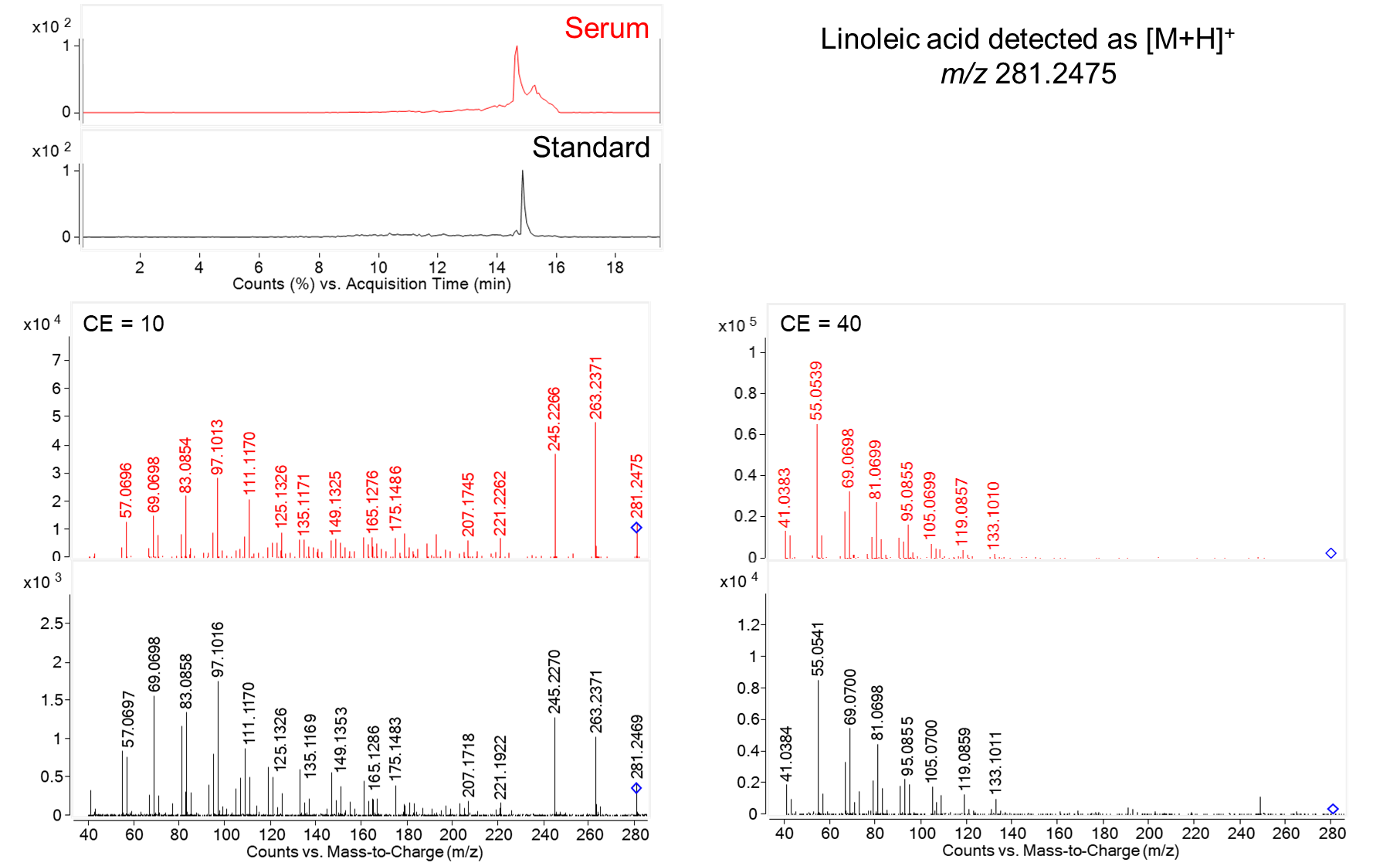
Figure S3d**. **Linoleic acid detected as [M+H]^+^ at *m/z* 281.2475.** **Top**) Chromatographic retention time was matched between synthetic standard and the endogenous molecule detected in serum. **Bottom**) Collision-induced dissociation product ion spectra comparison for synthetic and endogenous molecules at collision energy values of 10 and 40.

**
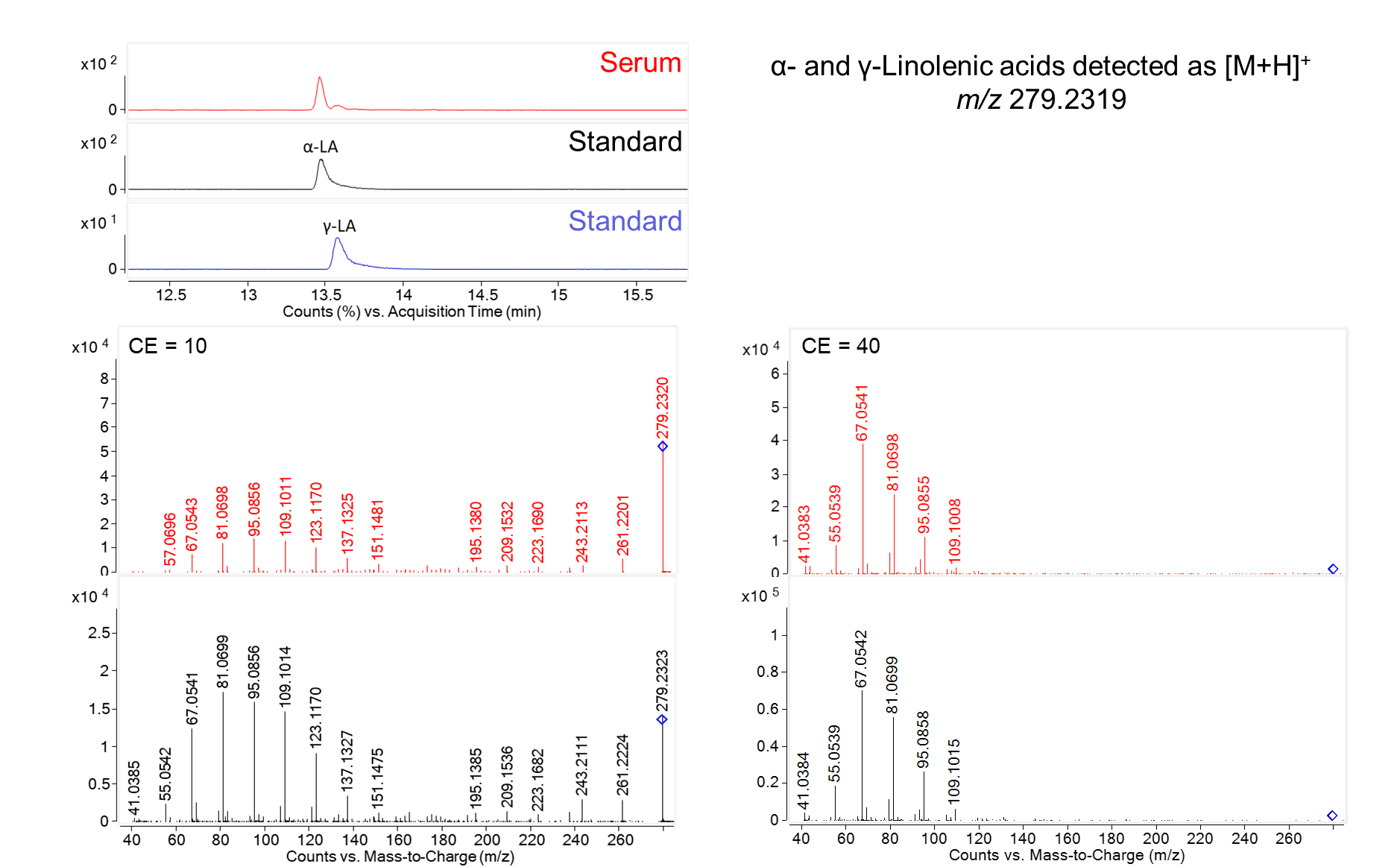
Figure S3e**. **α- and γ-Linolenic acids detected as [M+H]^+^ at *m/z* 279.2319.** **Top**) Chromatographic retention time was matched between synthetic standards and the endogenous molecules detected in serum. **Bottom**) Collision-induced dissociation product ion spectra comparison for synthetic and endogenous molecules at collision energy values of 10 and 40.

**
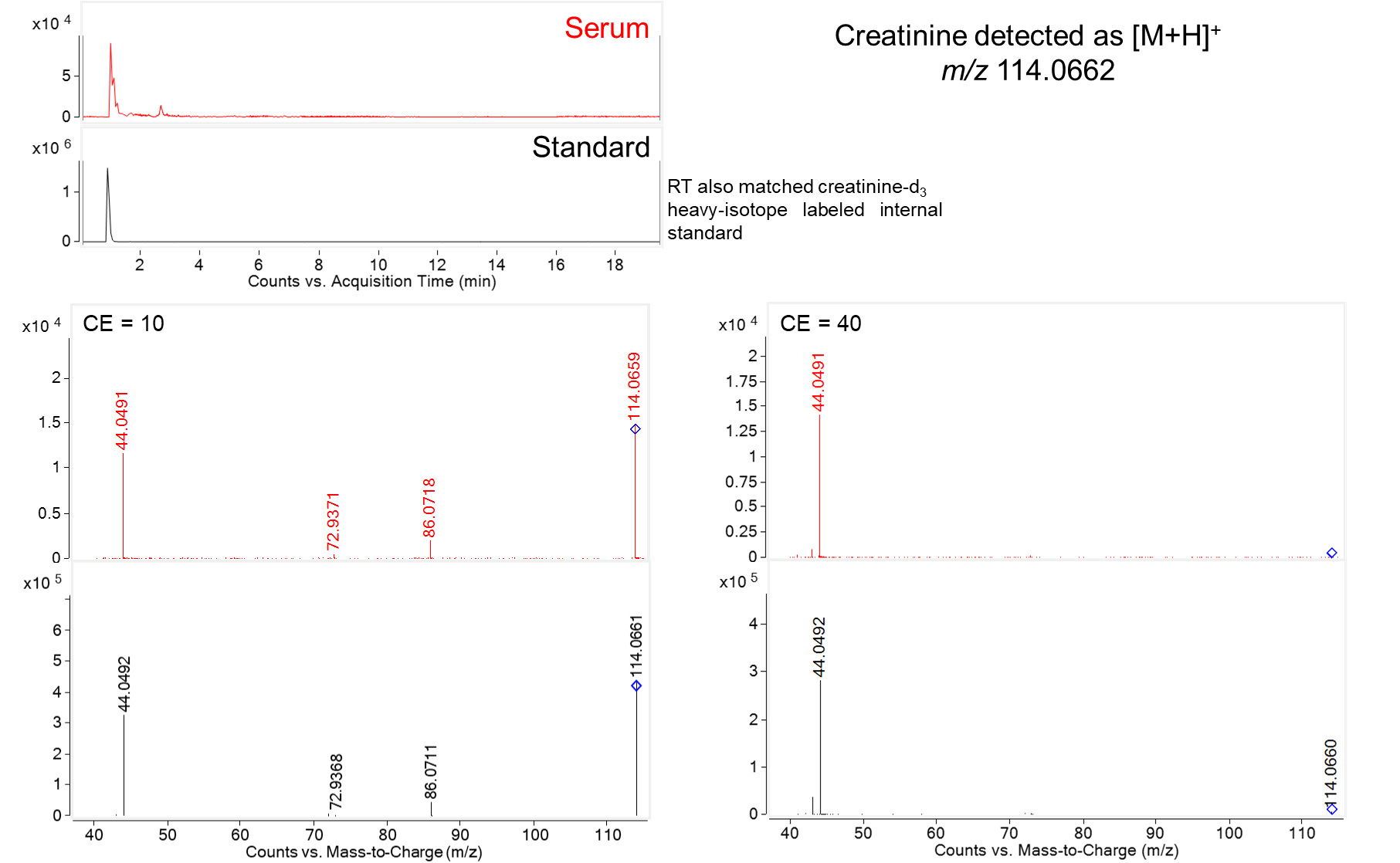
Figure S3f**. **Creatinine detected as [M+H]^+^ at *m/z* 114.0662.** **Top**) Chromatographic retention time was matched between synthetic standard and the endogenous molecule detected in serum. **Bottom**) Collision-induced dissociation product ion spectra comparison for synthetic and endogenous molecules at collision energy values of 10 and 40.

**
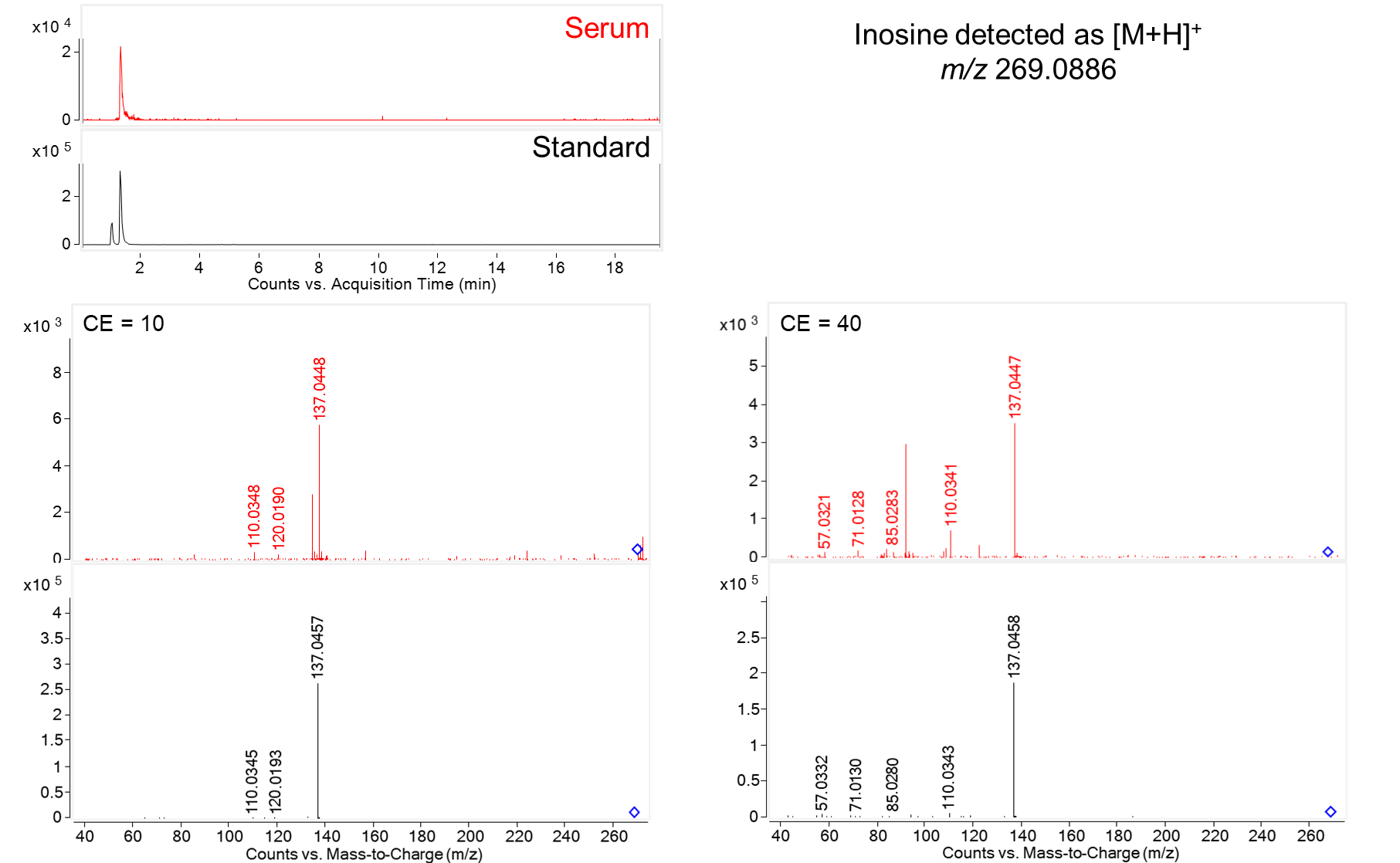
Figure S3g**. **Inosine detected as [M+H]^+^ at *m/z* 269.0886.** **Top**) Chromatographic retention time was matched between synthetic standard and the endogenous molecule detected in serum. **Bottom**) Collision-induced dissociation product ion spectra comparison for synthetic and endogenous molecules at collision energy values of 10 and 40.

**
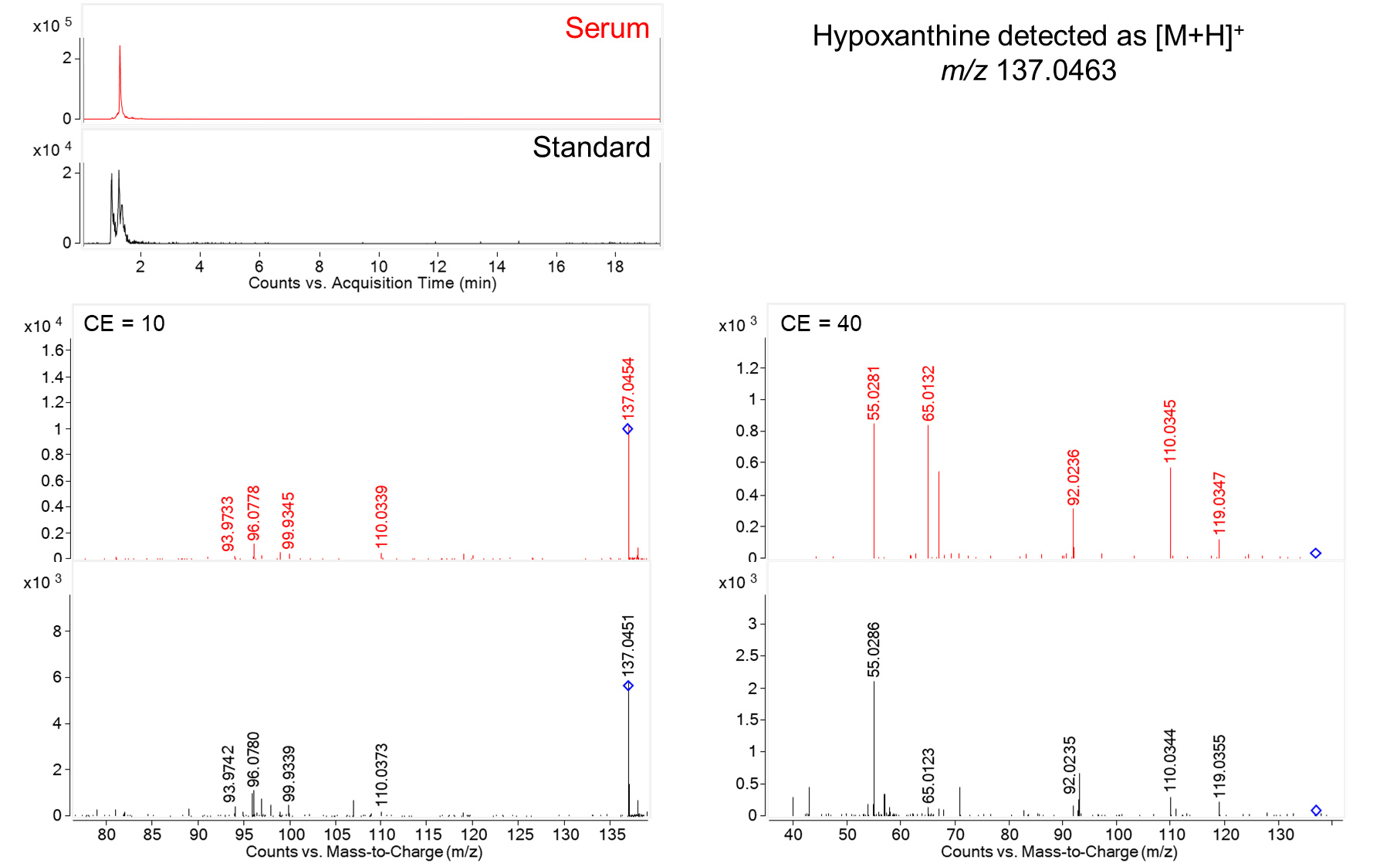
Figure S3h**. **Hypoxanthine detected as [M+H]^+^ at *m/z* 137.0463.** **Top**) Chromatographic retention time was matched between synthetic standard and the endogenous molecule detected in serum. **Bottom**) Collision-induced dissociation product ion spectra comparison for synthetic and endogenous molecules at collision energy values of 10 and 40.

**
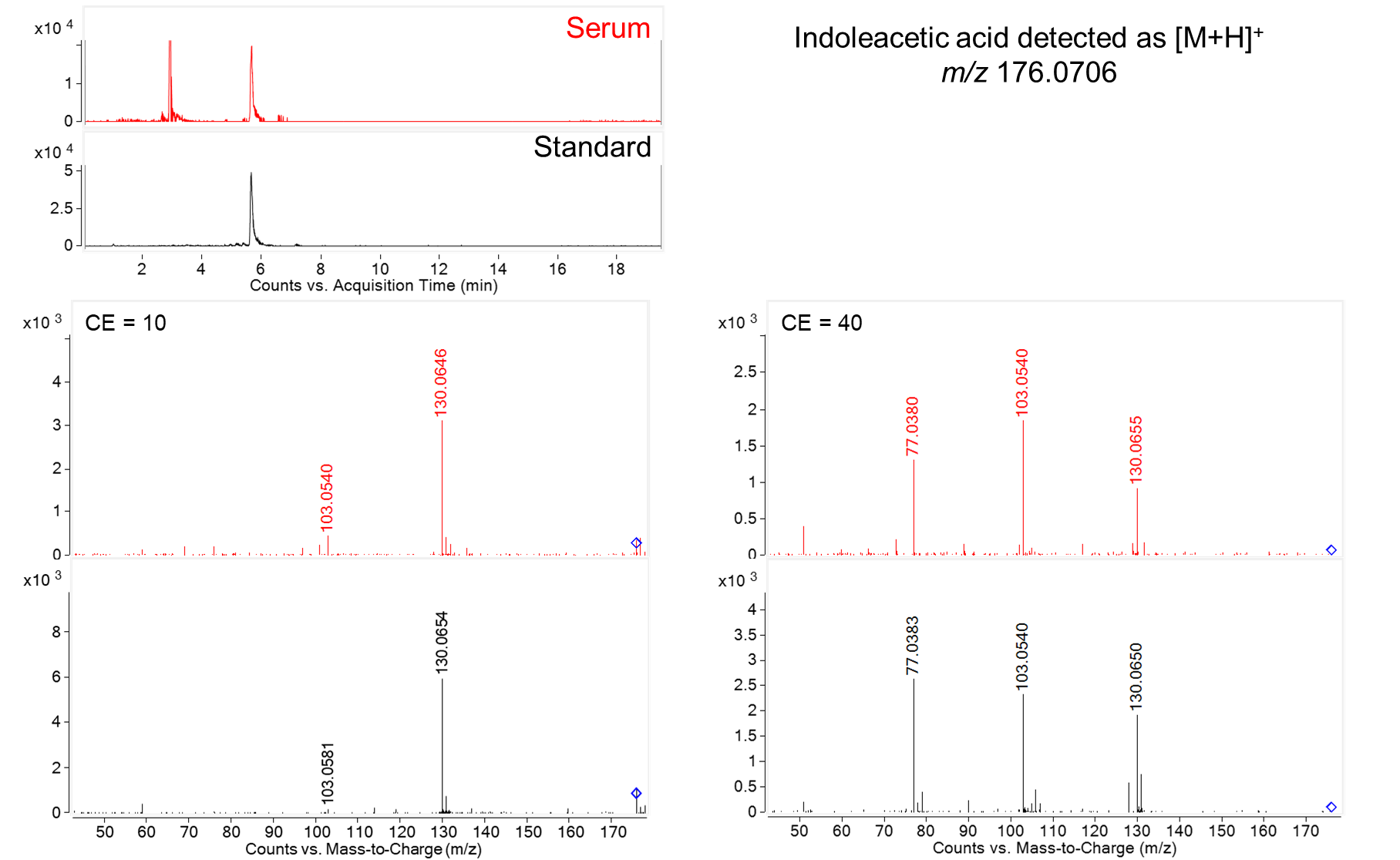
Figure S3i**. **Indoleacetic acid detected as [M+H]^+^ at *m/z* 176.0706.** **Top**) Chromatographic retention time was matched between synthetic standard and the endogenous molecule detected in serum. **Bottom**) Collision-induced dissociation product ion spectra comparison for synthetic and endogenous molecules at collision energy values of 10 and 40.

**
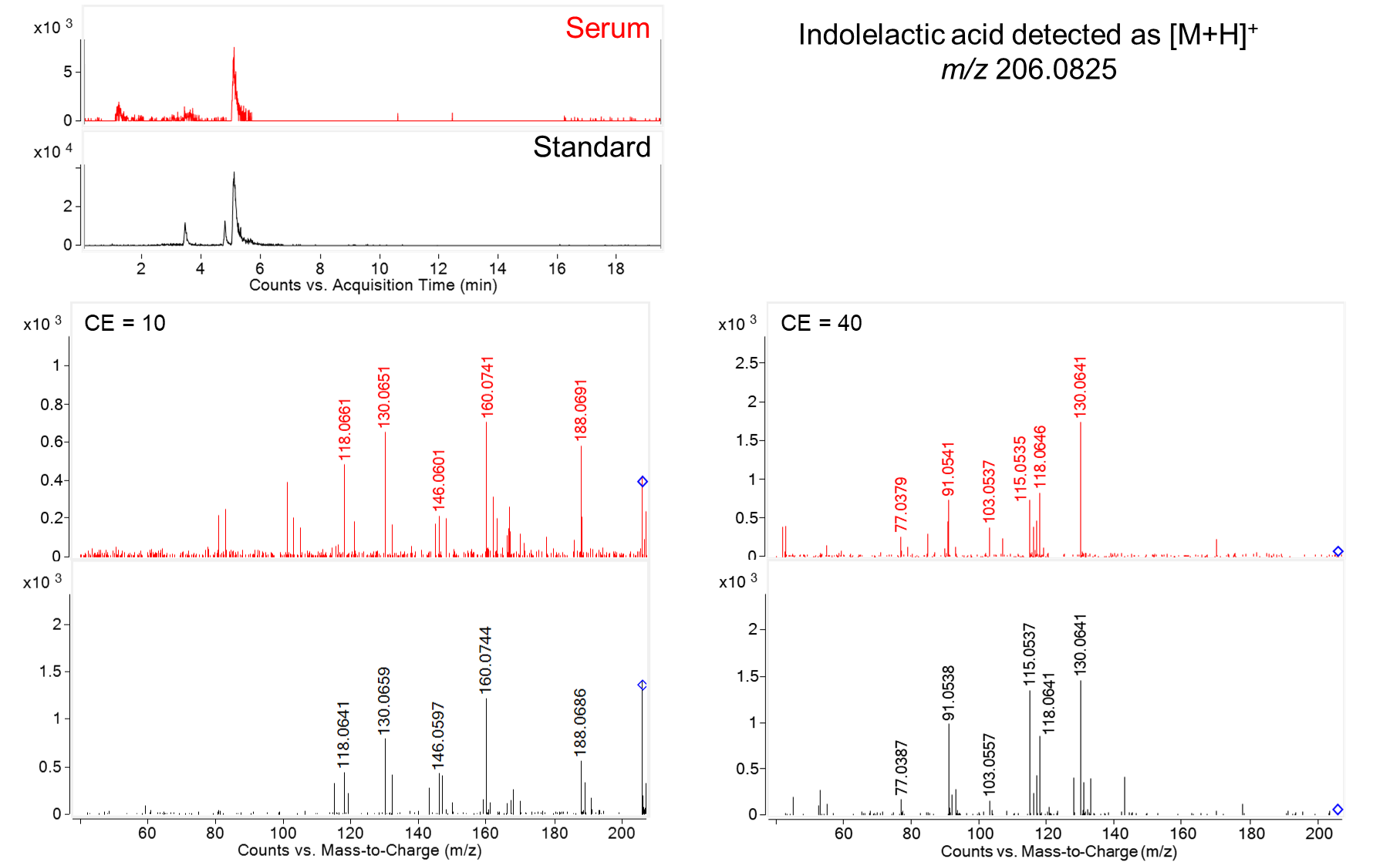
Figure S3j**. **Indolelactic acid detected as [M+H]^+^ at *m/z* 206.0825.** **Top**) Chromatographic retention time was matched between synthetic standard and the endogenous molecule detected in serum. **Bottom**) Collision-induced dissociation product ion spectra comparison for synthetic and endogenous molecules at collision energy values of 10 and 40.

**
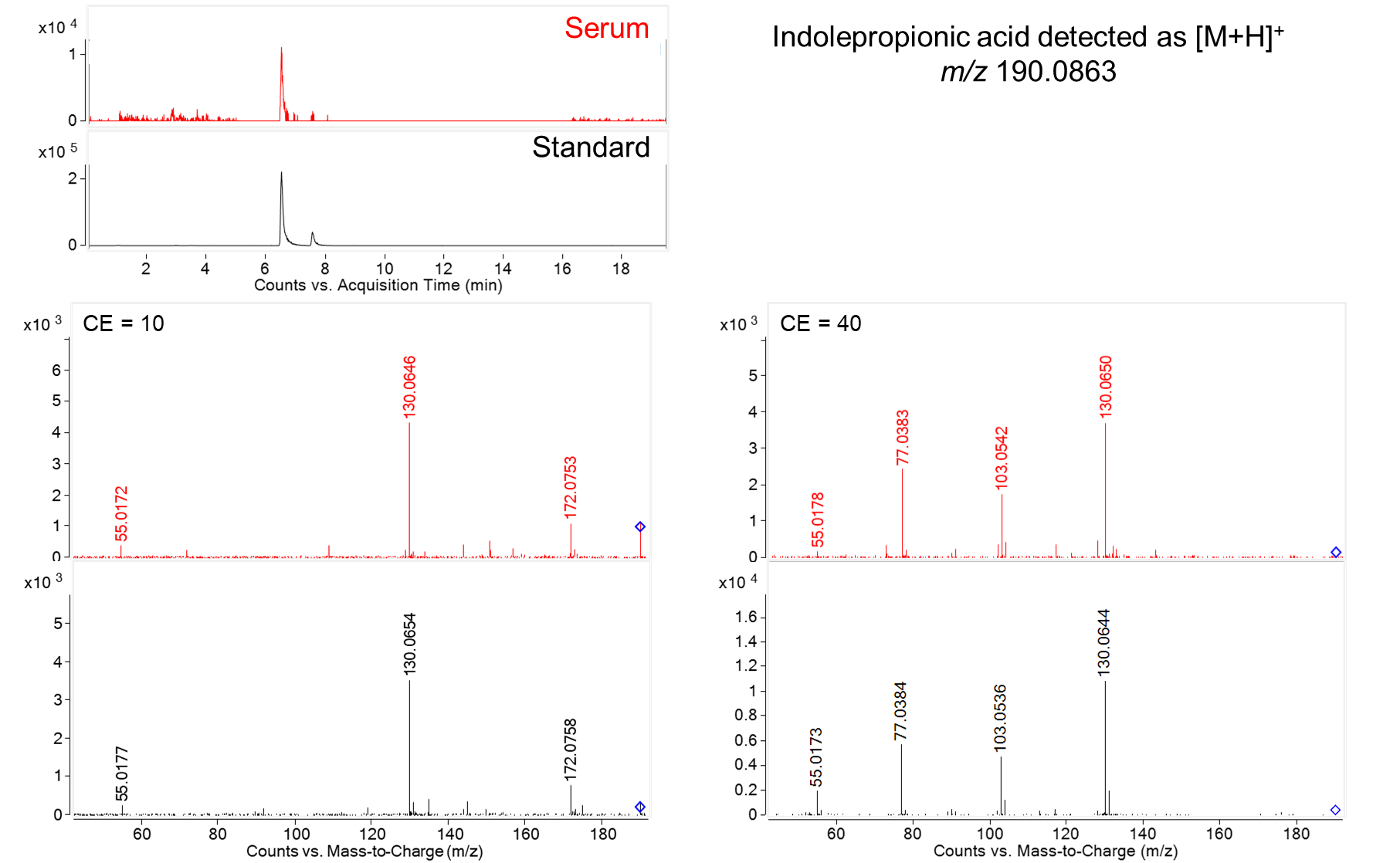
Figure S3k**. **Indolepropionic acid detected as [M+H]^+^ at *m/z* 190.0863. Top**) Chromatographic retention time was matched between synthetic standard and the endogenous molecule detected in serum. **Bottom**) Collision-induced dissociation product ion spectra comparison for synthetic and endogenous molecules at collision energy values of 10 and 40.

**
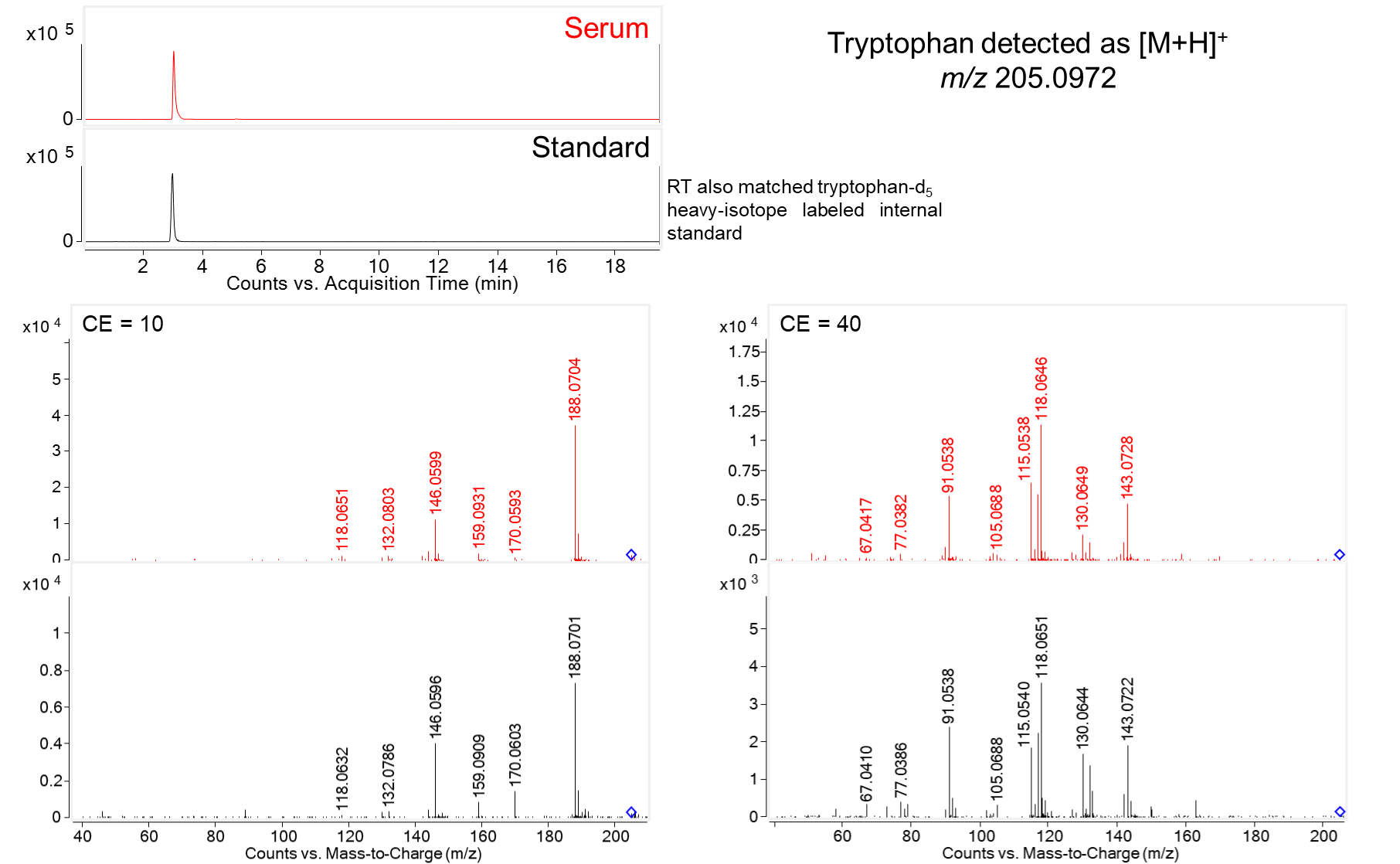
Figure S3l**. **Tryptophan detected as [M+H]^+^ at *m/z* 205.0972**. **Top**) Chromatographic retention time was matched between synthetic standard and the endogenous molecule detected in serum. **Bottom**) Collision-induced dissociation product ion spectra comparison for synthetic and endogenous molecules at collision energy values of 10 and 40.

**
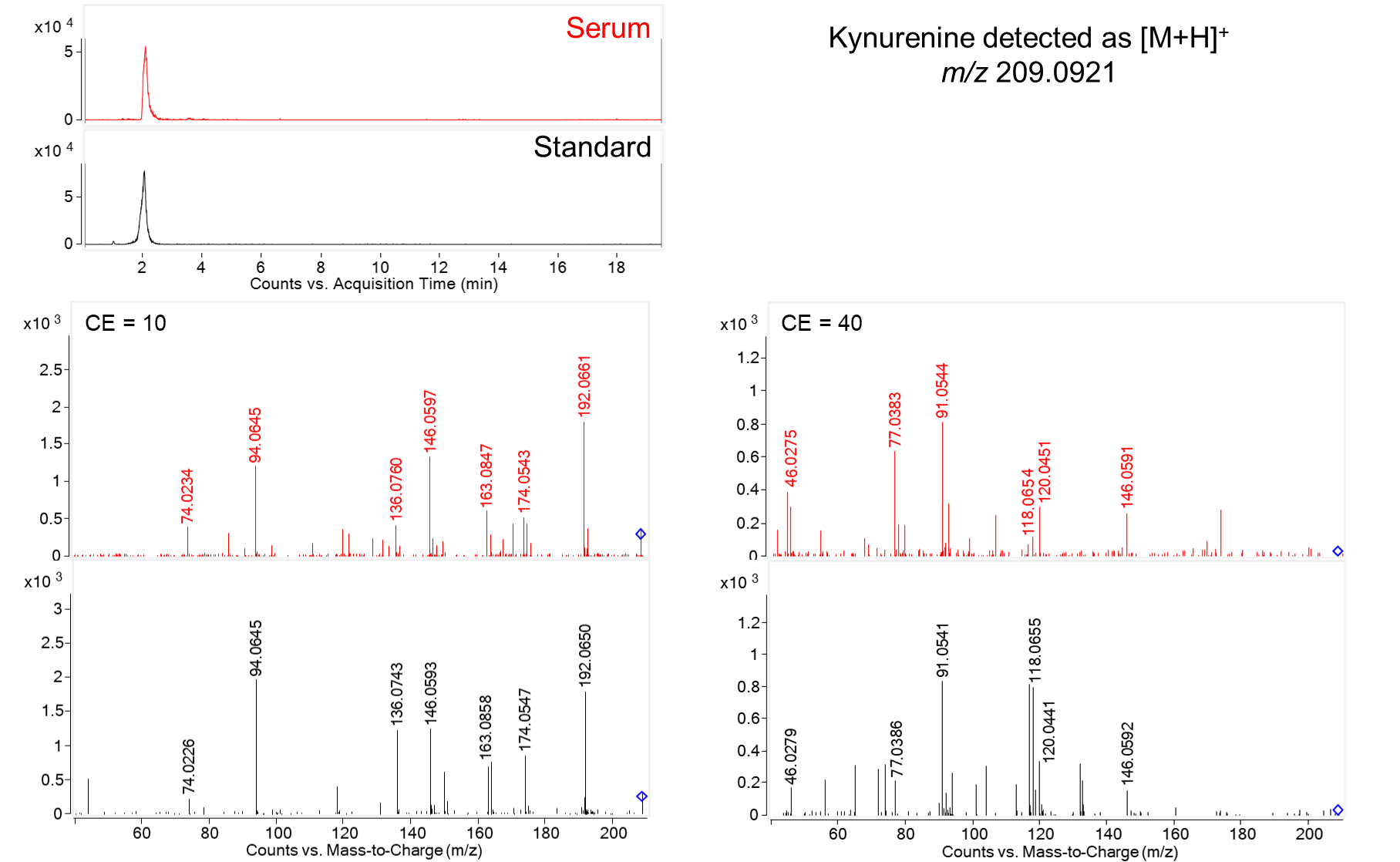
Figure S3m**. **Kynurenine detected as [M+H]^+^ at *m/z* 209.0921.** **Top**) Chromatographic retention time was matched between synthetic standard and the endogenous molecule detected in serum. **Bottom**) Collision-induced dissociation product ion spectra comparison for synthetic and endogenous molecules at collision energy values of 10 and 40.

**
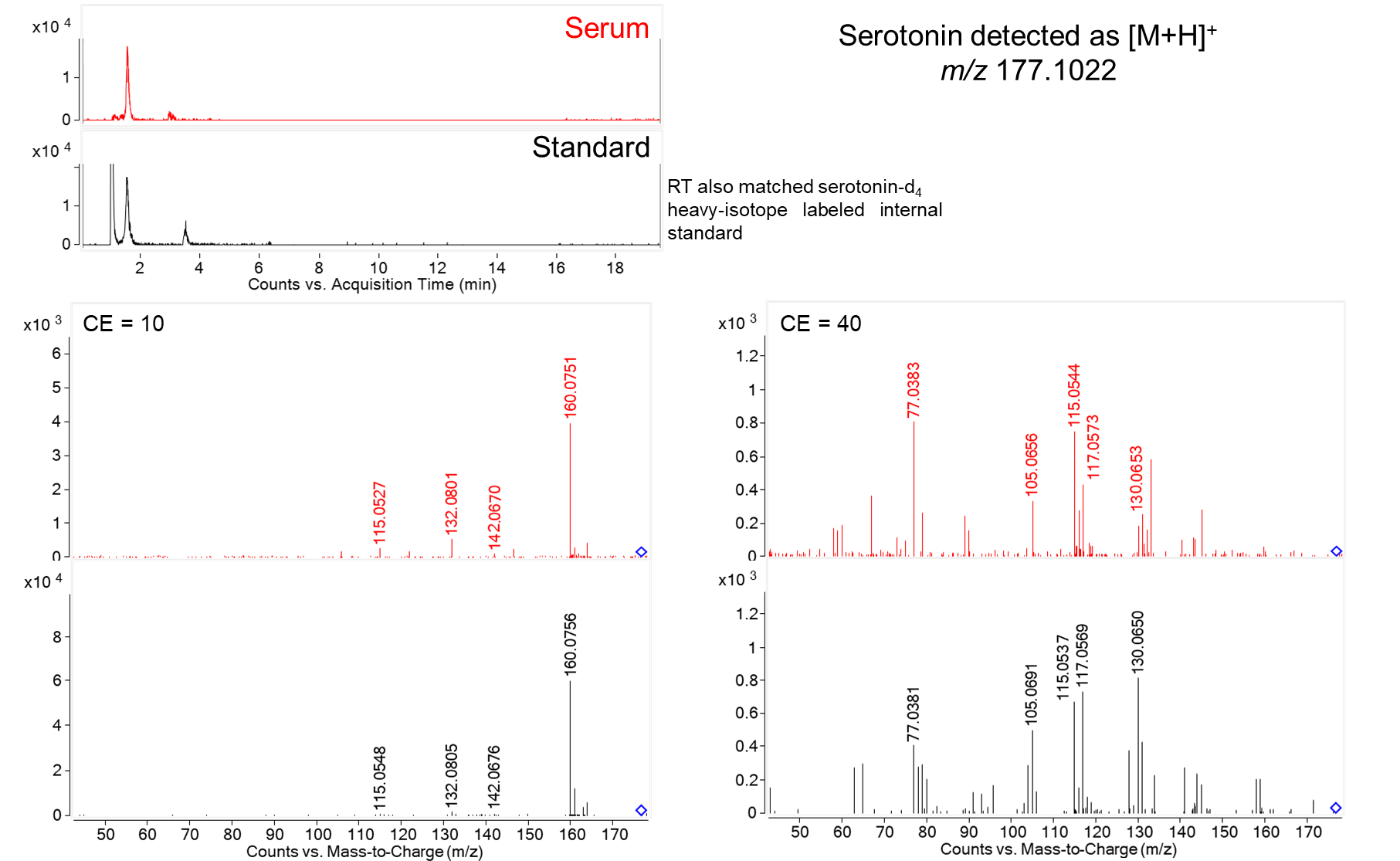
Figure S4n**. **Serotonin detected as [M+H]^+^ at *m/z* 177.1022**. **Top**) Chromatographic retention time was matched between synthetic standard and the endogenous molecule detected in serum. **Bottom**) Collision-induced dissociation product ion spectra comparison for synthetic and endogenous molecules at collision energy values of 10 and 40.

*
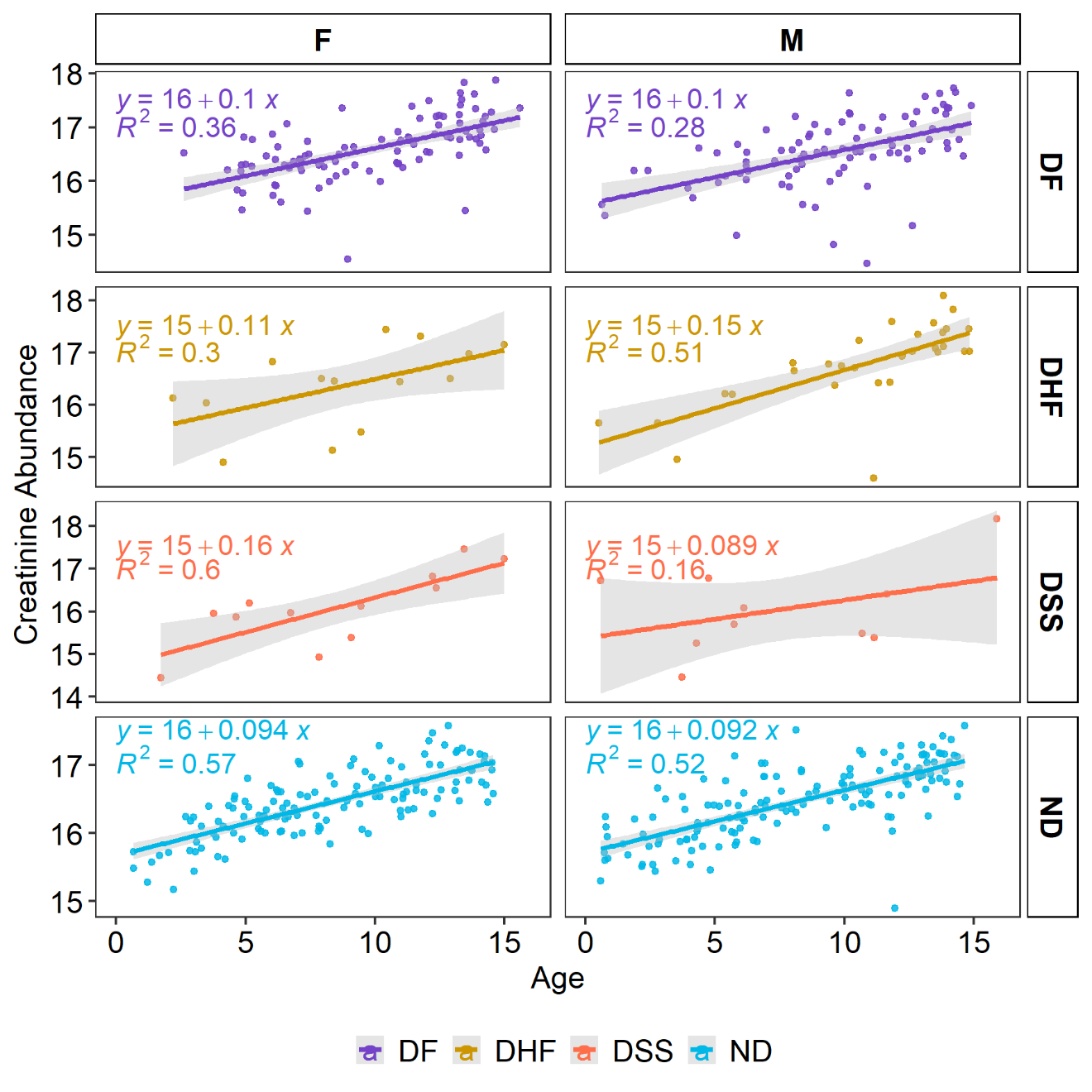
***Figure S4.** **Linear regressions for creatinine abundance as a function of patient age for each combination of patient sex and disease state.** The positive correlation between creatinine abundance and age is independent of patient sex and disease outcome.


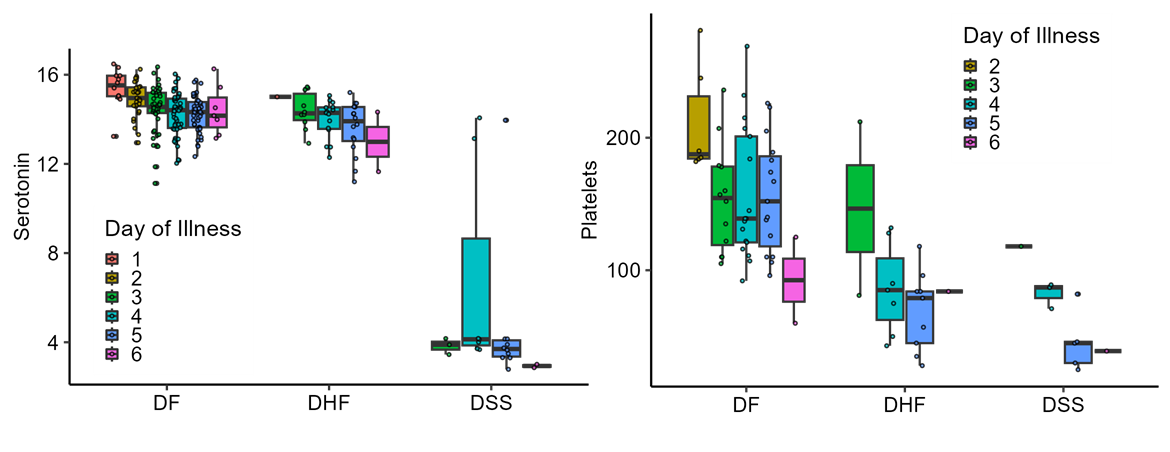


**Figure S5. Cross-sectional analysis of dengue patients by day of illness across different disease states**. Left) Serum serotonin abundance as a function of days of illness plotted for DF, DHF and DSS patients. Right) Serum platelets for a subset of 144 patients as a function of day of illness plotted for DF, DHF and DSS patents.

**
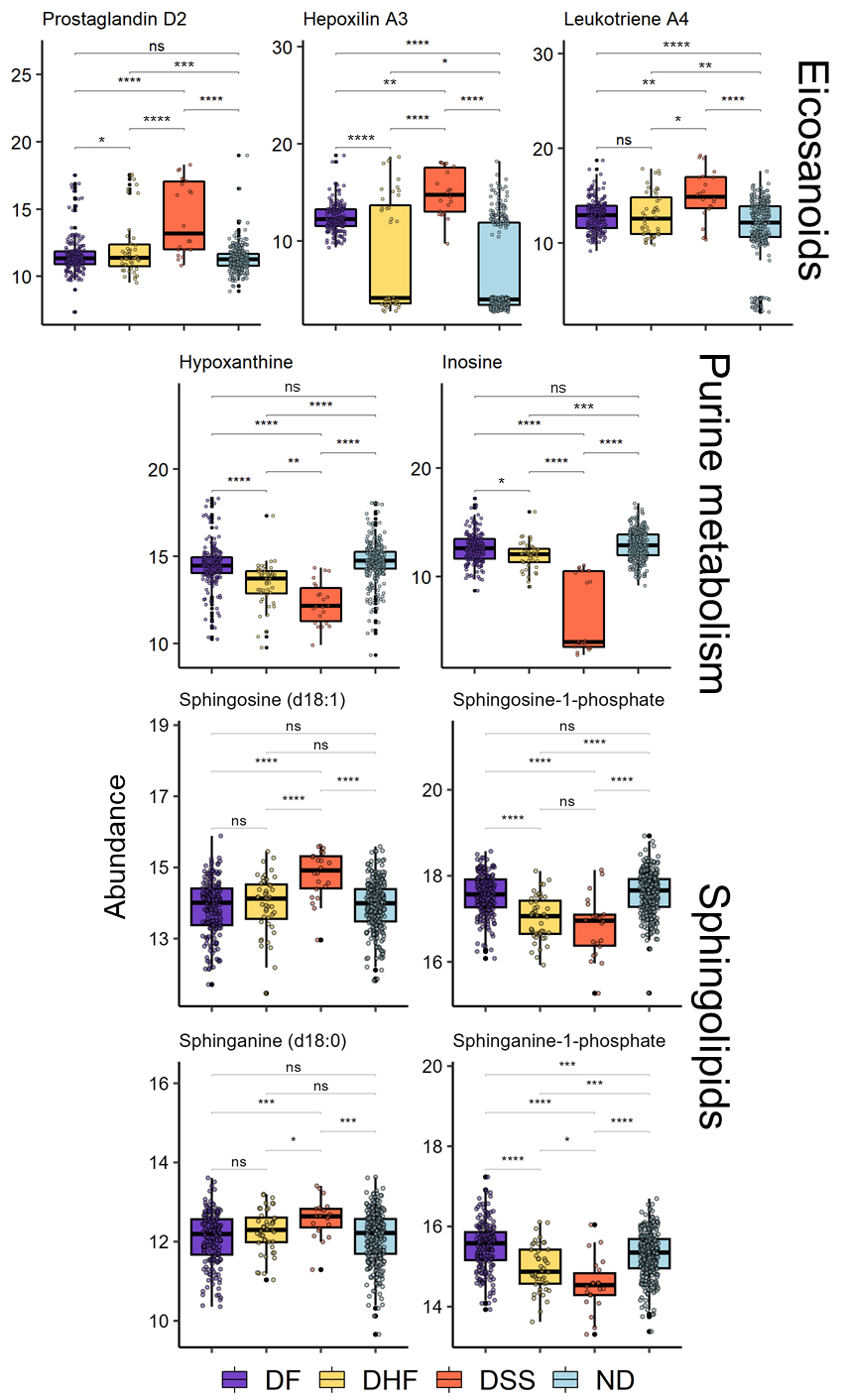
**

**Figure S6.** **Abundance of n-6 derived eicosanoids, molecules related to purine metabolism and sphingolipids in sera of ND and dengue patients.** Abundance is represented as the normalized and log2-transformed LC-MS peak area. Benjamini-Hochberg adjusted p-values generated using a moderated t-test were used to define statistical significance level; p < 0.0001, 0.001, 0.01, 0.05 were each represented by ****, ***, **, or *, respectively.


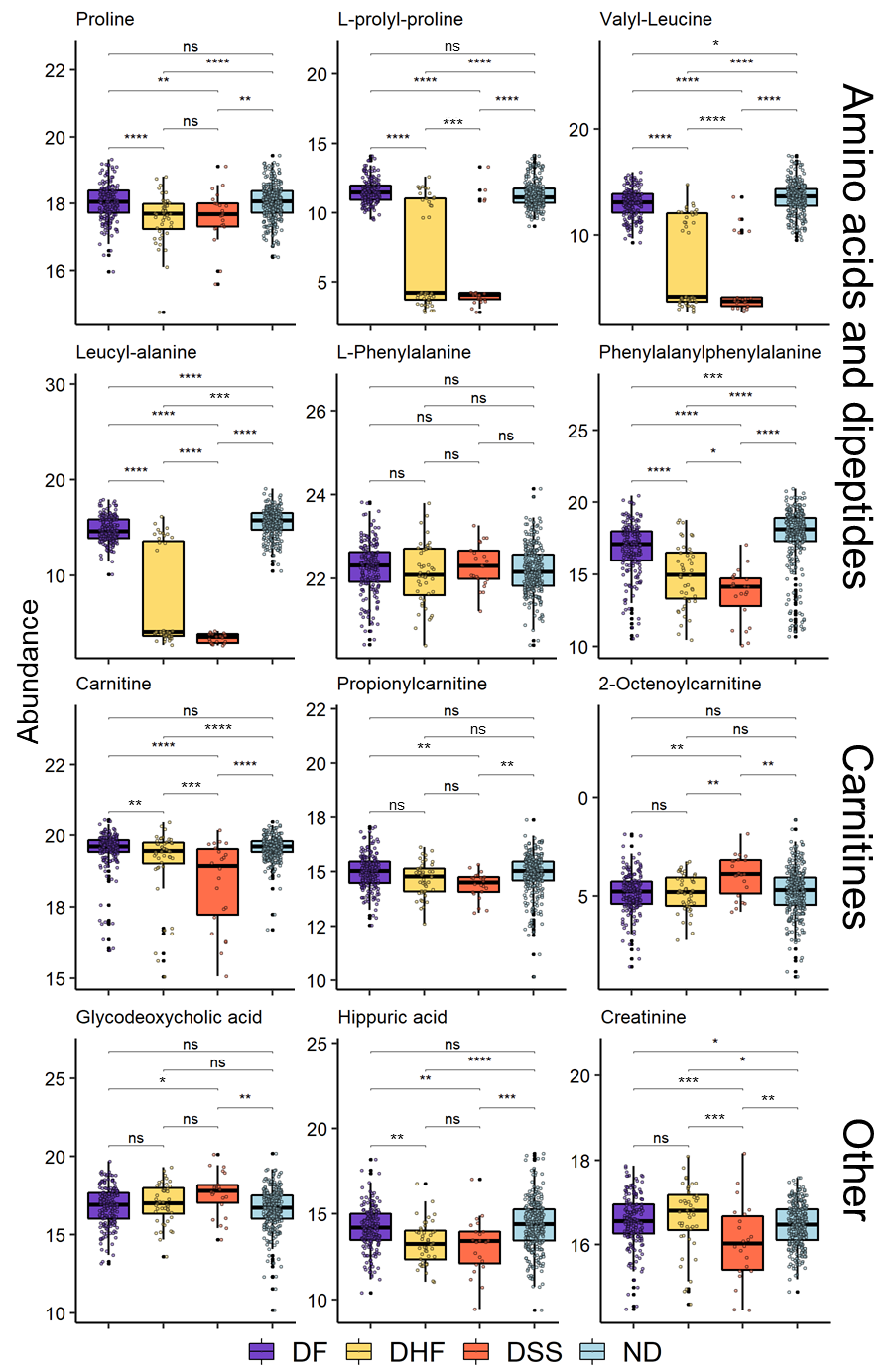


**Figure S7. Abundance of amino acids, dipeptides, carnitines and other metabolites in sera of ND and dengue patients.** Abundance is represented as the normalized and log2-transformed LC-MS peak area. Benjamini-Hochberg adjusted p-values generated using a moderated t-test were used to define statistical significance level; p < 0.0001, 0.001, 0.01, 0.05 were each represented by ****, ***, **, or *, respectively.

**
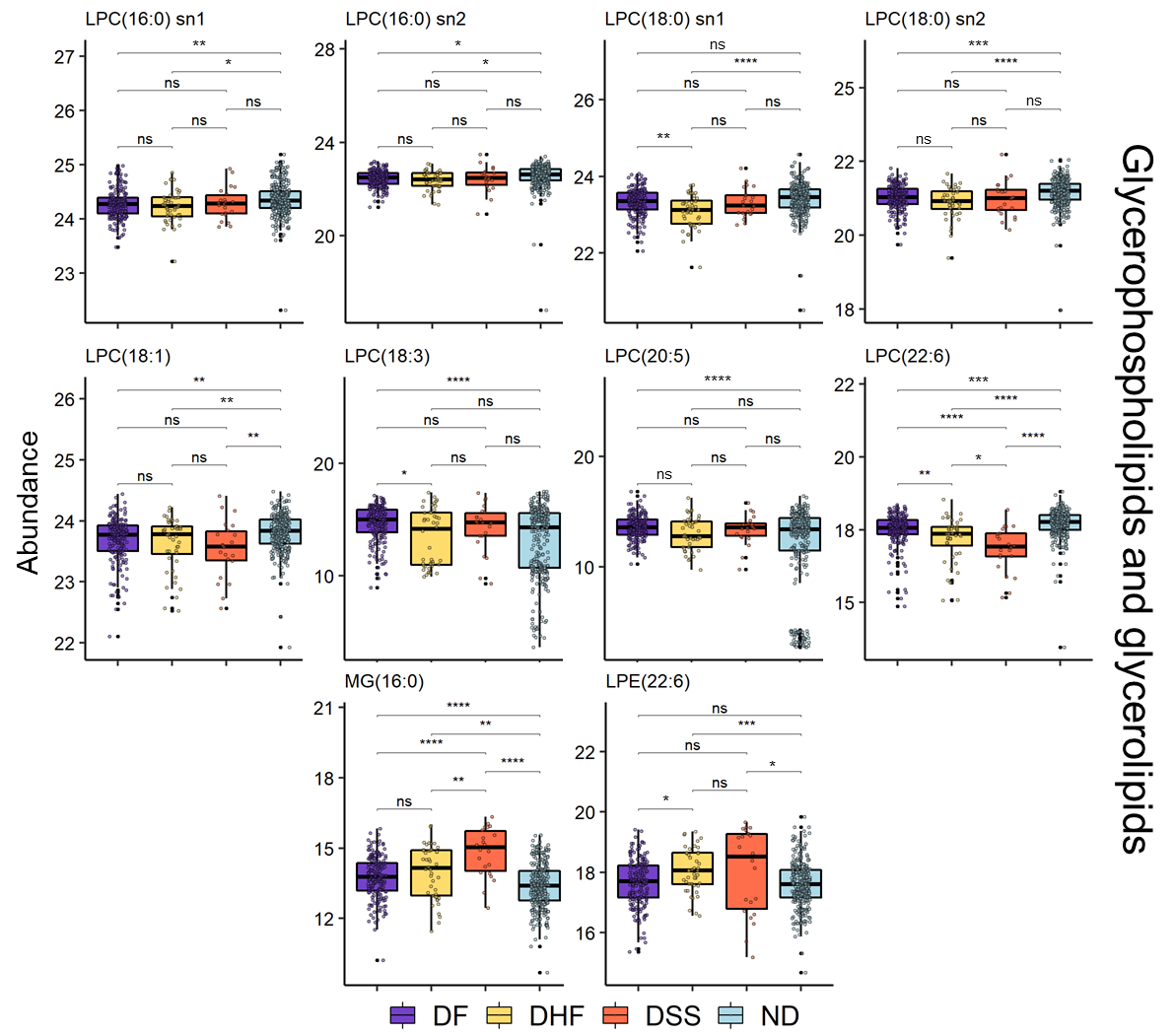
Figure S8. Abundance of glycerophospholipids and glycerolipids in sera of ND and dengue patients.** Abundance is represented as the normalized and log2-transformed LC-MS peak area. Benjamini-Hochberg adjusted p-values generated using a moderated t-test were used to define statistical significance level; p < 0.0001, 0.001, 0.01, 0.05 were each represented by ****, ***, **, or *, respectively.
